# CFTR mutation enhances Dishevelled degradation and results in impairment of Wnt-dependent hematopoiesis

**DOI:** 10.1038/s41419-018-0311-9

**Published:** 2018-02-15

**Authors:** Huaqin Sun, Yan Wang, Jieting Zhang, Yan Chen, Yanyan Liu, Ziyuan Lin, Mingfeng Liu, Kai Sheng, Huijuan Liao, Kam Sze Tsang, Xiaohu Zhang, Xiaohua Jiang, Wenming Xu, Meng Mao, Hsiao Chang Chan

**Affiliations:** 1 SCU-CUHK Joint Laboratory for Reproductive Medicine, Key Laboratory of Birth Defects and Related Diseases of Women and Children (Sichuan University), Ministry of Education, Department of Pediatrics, West China Second University Hospital, Sichuan University, 610041 Chengdu, China; 2 Epithelial Cell Biology Research Center, School of Biomedical Sciences, Faculty of Medicine, The Chinese University of Hong Kong, Hong Kong SAR, China; 3 Prenatal Diagnosis Center, Department of Obstetrics & Gynecologic, West China Second University Hospital, Sichuan University, 610041 Chengdu, China; 4 Department of Anatomical and Cellular Pathology, Faculty of Medicine, The Chinese University of Hong Kong, Hong Kong, China

## Abstract

Mutations of cystic fibrosis transmembrane conductance regulator (CFTR) cause cystic fibrosis (CF) with a multitude of clinical manifestations. Some CF patients develop clinically significant anemia, suggesting that CFTR may regulate hematopoiesis. Here, we report that *cftr* mutant zebrafish model exhibits primitive and definitive hematopoietic defects with impaired Wnt signaling. Cftr is found to interact, via its PDZ-binding domain (PDZBD), with Dishevelled (Dvl), a key component of Wnt signaling required for hematopoietic progenitor specification, thus protecting Dvl from Dapper1 (Dpr1)-induced lysosomal degradation. Defective hematopoiesis and impaired Wnt signaling in *cftr* mutant can be rescued by overexpression of wild-type or channel function-defective G551D mutant CFTR with an intact PDZBD, but not Cftr with mutations in the PDZBD. Analysis of human database (http://r2.amc.nl) shows that CFTR is positively correlated with DVL2 and Wnt-related hematopoietic factors in human blood system. The results reveal a previously unrecognized role of CFTR, which is independent of its channel function, in regulating DVL degradation and thus Wnt signaling required for hematopoiesis in both zebrafish and humans, providing an explanation for the anemic phenotype of CF patients.

## Introduction

Cystic fibrosis transmembrane conductance regulator (CFTR) is an ATP-binding cassette (ABC) transporter superfamily cAMP-activated anion channel^1^. It was first found to be expressed in a wide variety of epithelial tissues^2^. Mutations of the CFTR have been shown to cause cystic fibrosis (CF), the most common lethal genetic disease in Caucasians with a hallmark defect in electrolyte and fluid transport affecting multiple organ systems with a multitude of clinical manifestations^3,4^. Defective CFTR ion channel function due to the most common mutation of CFTR, ΔF508 mutation, has been shown to underline the pathogenesis of some of the disease conditions in CF, such as obstructive lung disease^5,6^, pancreas exocrine deficiency^7^, CF-related diabetes^8^, abnormal gonad function, and infertility^9–11^. However, many CF symptoms cannot be explained by a disease mechanism based on channelopathy.

Accumulating evidence has indicated that, in addition to its channel function, CFTR may also act as a potential regulator via its interaction with a large number of proteins^12^. Notably, CFTR has a PDZ-binding domain (PDZBD) at its carboxy terminus, which can bind to proteins with PDZ domain^13^, the most abundant protein interaction module in the human genome^14^. Our previous study has found that CFTR interacts with adherens junction molecule AF-6/afadin via PDZBD, thus regulating epithelial polarity and affecting cancer metastasis^15^. Ruan et al. have recently demonstrated that CFTR interacts with ZO-1 through PDZBD, and modulates the expression of ZO-1-ZONAB pathway downstream genes in cell proliferation and differentiation^16^. Of note, this effect was observed in the absence of cAMP stimulation, a condition that keeps CFTR channel open probability near zero, suggesting that the channel activity of CFTR is not required for its role in tight junction assembly. However, whether CFTR channel activity is required for its regulatory role has not been rigorously tested. Nevertheless, the demonstrated ability of CFTR to interact with an array of proteins and regulate different cellular processes has shed new light on the pathogenesis of myriad clinical manifestations of CF, which cannot be explained simply by a defect in CFTR channel function. For example, severe anemia in CF has also been reported in 4% of CF infants^17^. This suggests possible and yet unexplored involvement of CFTR in hematopoiesis.

Wnt/β-catenin signaling plays a major role in hematopoiesis^18^. Dishevelled (Dvl) is a crucial adaptor protein, which also contains a PDZ domain, in the canonical Wnt signaling pathway that leads to the nuclear translocation of β-catenin. Wnt signaling ultimately activates the Cdx-Hox pathway, which drives the expression of key transcription factors, such as *scl* and *gata1a*, that are central to the specification of hematopoietic precursors^19,20^.

Controlling Dvl protein stability is an important mechanism for the cells to regulate the Wnt signaling pathway. Dvl is degraded through either the proteasomal or lysosomal pathway^21^. Several proteins have been reported to induce Dvl proteasomal degradation^21–23^, whereas the study on Dvl lysosomal degradation is less extensive. Dapper1 (DPR1) has been identified as a Dvl-interacting protein that promotes Dvl turnover through lysosomes^24^. Specifically, DPR1 enhances the interaction of Dvl2 with the Von Hippel–Lindau tumor suppressor (pVHL), an E3 ubiquitin ligase component, resulting in Dvl2 ubiquitination and degradation in lysosomes^25^.

The zebrafish has emerged to be an ideal model organism for hematopoiesis research owing to a number of experimental advantages, including short lifespan, external development, transparent embryos, and genetic amenability^26^. Similar to other vertebrate organisms, zebrafish also have two waves of hematopoiesis, which occur in a spatially unique manner^27^. Zebrafish primitive hematopoiesis takes place in ventrolateral plate mesoderm-derived tissue called intermediate cell mass (ICM), which gives rise to erythrocytes in the posterior part of the embryo and generates myeloid cells in the anterior part of the embryo^28–30^. After primitive hematopoiesis, definitive hematopoietic stem cells (HSCs) emerge from the ventral wall of dorsal aorta in a region known as aorta-gonad-mesonephros (AGM)^30^, and migrate to the posterior region in the tail called the caudal hematopoietic tissue (CHT), before seeding the kidney marrow, which is equivalent to bone marrow in mammals, where HSCs produce blood cells of all lineages for the rest of the lifespan^28,29^. Importantly, the programs controlling hematopoiesis in zebrafish, including Wnt signaling, are conserved in mammals including humans, making zebrafish a clinically relevant model system^26^.

Given the clinical incidences of anemia in CF patients and the importance of Wnt signaling in hematopoiesis, we hypothesized that CFTR might regulate Wnt-dependent hematopoiesis through a potential interaction between Cftr and Dvl via their PDZBD/PDZ domain. We undertook the present study to test this hypothesis using zebrafish model, and revealed a previously unrecognized role of CFTR, which is independent of its channel function, in the Wnt signaling pathway crucial for both primitive and definitive hematopoiesis, providing an explanation as to how CFTR mutation may lead to anemia in CF patients.

## Results

### cftr mutant leads to primitive and definitive hematopoietic deficiency in zebrafish

**Fig. 1:**
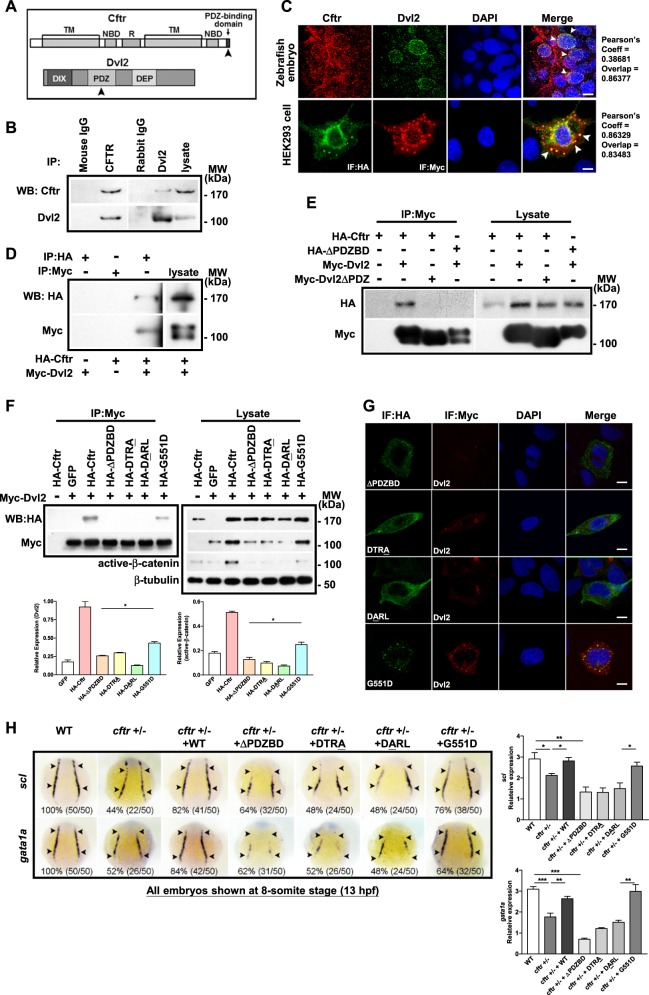
Cftr PDZBD but not its channel function is required for its interaction with Dvl2 and hematopoiesis. **a** Schematic drawing of PDZBD in Cftr and PDZ domain in Dvl2. Referring to Gee, H. Y. (2011)[57] and Wallingford, J. B. (2005)[58]. NBD nucleotide-binding domain, TM transmembrane domain, R regulatory domain, DIX Dishevelled and AXIN domain, DEP Dishevelled EGL-10 Pleckstrin domain. **b** Co-immunoprecipitation (Co-IP) of endogenous Dvl2 and Cftr in zebrafish embryos. **c** Co-localization of Cftr and Dvl2 in zebrafish embryo and HEK293 cells. Arrowheads indicate co-localized sites. Scale bar 5 μm. **d** In vitro binding assay identifies the physical interaction of Cftr with Dvl2. **e** Co-IP of exogenous Cftr and Dvl2 in HEK293 cells showing lack of interaction when Cftr PDZBD or Dvl2 PDZ domain is deleted. **f** Co-IP showing the interaction of Dvl2 with G551D, but not Cftr PDZBD mutants (either deletion or point mutation), and western blotting showing that Cftr and G551D overexpression increased the expression of Dvl2 and active-β-catenin more significantly than that induced by Cftr PDZBD mutants. **g** Immunofluorescence analysis in HEK293 cells shows that either of the CFTR PDZBD mutants is not co-localized with Dvl2, whereas overexpressed G551D is co-localized with Dvl2. **h** Effects of different *cftr* mutants mRNA in rescuing hematopoietic defects in *cftr* mutant zebrafish embryos. Injection of *cftr* PDZBD mutants individually, with deletion or point mutation, could not rescue the hematopoietic defect in *cftr* mutant embryos. Injection of G551D rescues the hematopoietic defect in *cftr* mutant embryos. Embryos shown are dorsal views with anterior to the top at 8-somite stage (13 hpf)

Zebrafish Cftr, similar to human CFTR^31,32^, contains a PDZBD at its carboxyl terminus (Appendix Fig. S1; see also Fig. 1a). Whole-mount in situ hybridization (WISH) detected both maternal and zygotic *cftr* expression throughout early development, with a relative enrichment in the branchial and pharyngeal arches at 24 hpf (prim-5 stage) (Appendix Fig. S2). This expression patterns suggest that Cftr plays a role in early axis formation in addition to previously reported Kupffer’s vesicle (KV) development^32^.

**Fig. 2:**
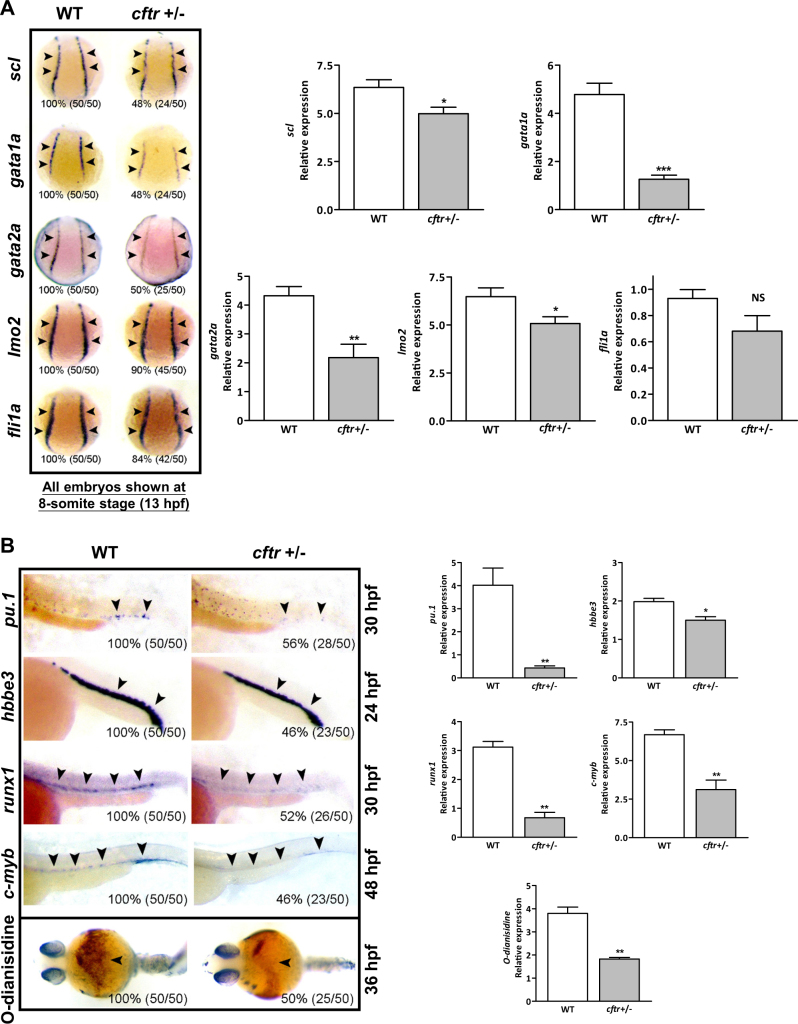
Cftr critically regulates hematopoiesis in zebrafish. **a** *cftr* mutants show hematopoietic defects at 8-somite stage (13 hpf). Embryos shown are dorsal views with anterior oriented at the top. **b** *cftr* mutants show hematopoietic defects at later stages. Embryos shown are lateral views with anterior to the left. Bottom panel: *cftr* mutants displayed decreased hemoglobin staining by O-dianisidine. Embryos shown are ventral views with anterior to the left. Arrowheads indicate the expression sites of each marker gene. All genes were assayed by WISH. Histogram representing the relative expression detected by signal strength grayscale using software ImageJ at corresponding assay. The percentage and numbers indicated in each picture are the ratio for the number (left in bracket) of affected embryos with phenotype similar to what is shown in the picture and the total number (right in bracket) of observed embryos. The same number labeling was used thereafter

We next generated a *cftr* frameshift mutant using TALENs technology^32^ (Appendix Fig. S3) and also observed the absent KV lumen phenotype at 8-somite stage in the mutant embryos as Navis et al. described^32^. Since a large percentage of homozygous *cftr* mutant larvae was lost beginning around 10 dpf^33^, we examined heterozygous *cftr* mutant instead throughout the study. At 8-somite stage (13 hpf), the early hematopoietic precursor markers *scl*, *gata2a*, and *lmo2* and the primitive erythroid marker *gata1a* were markedly reduced in *cftr* mutant embryos (Fig. 2a). In contrast, the early vascular development markers *fli1a* (Fig. 2a), lateral mesoderm marker, *draculin* (Appendix Fig. S4), and the later vascular endothelium marker, *flk1* (Appendix Fig. S5), were not changed significantly in *cftr* mutant. These results suggest a role of Cftr in zebrafish primitive hematopoiesis.

We next examined definitive hematopoiesis markers *runx1* (30 hpf) and *c-myb* (48 hpf) and found their expression in *cftr* mutants was markedly decreased (Fig. 2b). Furthermore, the expression of myeloid progenitor marker *pu.1* (30 hpf) and mature erythrocyte marker *hbbe3* (24 hpf) decreased dramatically in *cftr* mutant, suggesting that the development of erythroid and myeloid lineages was both impaired. Finally, the staining of O-dianisidine, a marker for red blood cells, was also reduced in *cftr* mutant embryos at 36 hpf (Fig. 2b, bottom row), further indicating a severe defect in red blood cell formation. In addition, we also observed deficient expression of progenitor cell marker, *scl*, erythroid marker, *gata1a*, and myeloid marker, *l-plastin*, in early larval stages (3 dpf) (Appendix Fig. S6). Similar hematopoietic defects were obtained using *cftr* antisense morpholino oligonucleotides (Appendix Figs. S6–10), which could be rescued by co-injection of human *CFTR* mRNA, demonstrating the specificity of the morpholinos and functional conservation of the CFTR between fish and human (Appendix Fig. S7). Furthermore, overexpressing zebrafish *cftr* mRNA led to the expansion of hematopoietic markers at 8-somite stage (Appendix Fig. S11). Collectively, these results provide evidence supporting that Cftr plays a critical and evolutionarily conserved role in primitive and definitive hematopoiesis.

### Cftr deficiency inhibits Wnt-dependent hematopoiesis

**Fig. 3:**
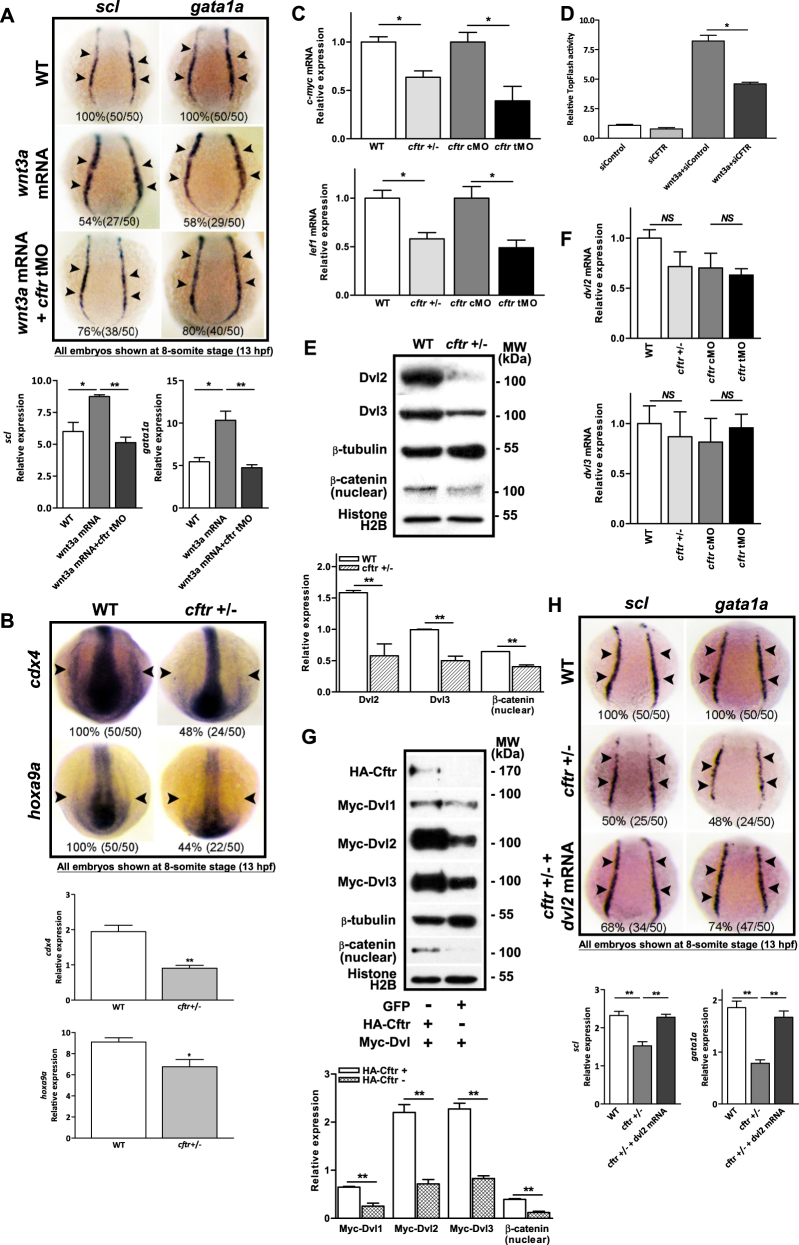
Involvement of Cftr in Wnt-dependent hematopoiesis in zebrafish. WISH showing impaired wnt3a-induced expression of *scl* and *gata1a* in *wnt3a* mRNA and *cftr* morpholino co-injected zebrafish (**a**) and decreased expression of *cdx4* and *hoxa9a* in *cftr* mutants (**b**). **c** Quantitative RT-PCR showing significantly reduced *c-myc* and *lef1* mRNA levels in *cftr* mutants. **d** CFTR knockdown by siRNA attenuated the Wnt reporter TopFlash activity in HEK293 cells (*n* = 3). **e** Western blot analysis showing reduced Dvl2, 3 and nuclear β-catenin expression in *cftr* mutants. **f** Quantitative RT-PCR showing no significant changes in *dvl2, 3* mRNA levels in *cftr* mutants. **g** Western blot analysis showing increased Dvl1-3 protein levels in co-expressing Cftr with Dvl1-3 HEK293 cells as compared to Dvl1-3 alone controls. **h** Effects of *dvl2* mRNA in rescuing hematopoietic defects in cftr mutants. Embryos shown in **a**, **b**, and **h** are dorsal views with anterior to the top at 8-somite stage (13 hpf)

Zebrafish hematopoietic progenitors emerge from the mesoderm at the gastrula stage^29^, and Wnt signaling plays a key role in this process^20^. We tested whether Cftr expression might affect Wnt-dependent hematopoiesis. Indeed, whereas injection of 1 pg of *wnt3a* mRNA led to an expansion of *scl* and *gata1a* in 54% and 58% of embryos, respectively, at the 8-somite stage (Fig. 3a), co-injection with equal amount of *cftr* tMO reduced the percentages of embryos with expansion of *scl* and *gata1a* to 24% and 20%, respectively (with 76% and 80% of embryos displaying no expansion of *scl* and *gata1a*, respectively, Fig. 3a). WISH results further revealed that the depletion of *cftr* either by TALENs or morpholinos also reduced the expression of *cdx4*, *hoxa9a*, *c-myc*, and *lef1* (Fig. 3b, c and Appendix Fig. S12), which are key Wnt targets known to play essential role in hematopoiesis^20,34^. We further examined the effect of CFTR knockdown, induced by siRNA, on the Wnt3a-induced TopFlash luciferase activity in HEK293 cells and observed a significant reduction (Fig. 3d), confirming a role of CFTR in regulating Wnt signaling. Taken together, these results suggest an important role of CFTR in Wnt-dependent hematopoiesis.

### Dvl is required for CFTR-regulated Wnt-dependent hematopoiesis

To further explore the role of Cftr in Wnt signaling during hematopoiesis, we further examined the effect of Cftr expression on key adaptors of the Wnt signaling, Dvls, in zebrafish. We found that the protein levels, but not mRNA level, of Dvl2 and Dvl3, were significantly reduced in *cftr* mutant and morphants at the beginning of gastrula period (5hpf) (Fig. 3e, f and Appendix Fig. S13). Furthermore, nuclear β-catenin was also significantly reduced in *cftr* mutant embryos or morphants (Fig. 3e and Appendix Fig. S13), consistent with a disruption of Wnt signaling. Similar to the findings in zebrafish, co-transfection of *HA-Cftr* significantly increased protein levels of Dvl1-3, as well as nuclear β-catenin, in HEK293 cells transfected with *myc*-*dvl1*-3 (Fig. 3g), suggesting that Cftr contributes to the maintenance of Dvl protein levels, thereby sustaining Wnt signaling activation. Thus, it appears plausible that the impaired hematopoiesis in *cftr* mutant or morphants could be due to a deficiency in Dvl. Indeed, injection with 30 pg of *dvl2* mRNA ameliorated the hematopoietic defect caused by deficient Cftr: percentages of embryos with normal *scl* and *gata1a* expression pattern increased from 50% to 68%, and from 52% and 74%, respectively, in *cftr* mutant (Fig. 3h), and from 44% to 74%, and from 24% to 88%, respectively, in *cftr* morphants (Appendix Fig. S14). Concomitantly, active-β-catenin and Wnt target genes, *c-myc* and *lef1*, also showed recovered expression pattern in *cftr* mutant embryos injected with *dvl2* mRNA (Appendix Fig. S15). Similar rescuing effects of *dvl2* mRNA on definitive hematopoiesis markers, *runx1* and *c-myb*, were also observed at 28 hpf and 48 hpf (Appendix Fig. S16). These data suggest that defective Cftr results in a Dvl deficiency, and thus, an impaired Wnt-dependent hematopoiesis.

### Cftr functionally interacts with Dvl via PDZBD

The reduced Dvl in *cftr* mutant or morphants suggested the importance of Cftr in stabilizing Dvl possibly through a direct protein–protein interaction. Reciprocal immunoprecipitation (co-IP) of endogenous Cftr and Dvl2 in zebrafish embryos at 5 hpf (Fig. 1a, b) and mouse bone marrow (Appendix Fig. S17), which consists of primarily hematopoietic cells, was conducted to confirm this hypothesis. Furthermore, whole-mount immunostaining assay in zebrafish embryos revealed co-localization of CFTR with Dvl2, with the majority of the signals detected in the cytoplasm (Fig. 1c). Similarly, exogenously expressed Cftr and Dvl2 were co-localized in HEK293 cells (Fig. 1c). In addition, the interaction between Cftr and Dvl2 was validated by in vitro protein binding assay (Fig. 1d).

To confirm the importance of PDZBD and PDZ domain in the interaction between Cftr and Dvl2, we constructed deletion mutants of Cftr and Dvl2 lacking PDZBD and PDZ domain, respectively. Co-IP in HEK293 cells showed that either mutation abrogated the interaction between these two proteins (Fig. 1e), suggesting the requirement of both PDZBD and PDZ for Cftr–Dvl2 interaction. To provide further evidence for the involvement of the PDZBD of Cftr in its interaction with Dvl2, we generated two additional mutants, DTRA and DARL, each carrying a point mutation in PDZBD as described by Moyer et al.^35^. Similar to Cftr PDZBD deletion mutant, both point mutations failed to bind to Dvl2 and enhance the expression of Dvl2 and active β-catenin in HEK293 cells (Fig. 1f, g). We next compared the ability of *cftr* WT and *cftr* PDZBD mutants to rescue the hematopoietic defect in *cftr* mutant embryos. Whereas injection with 50 pg of wild-type (WT) *cftr* mRNA increased percentages of *cftr* embryos with normal *scl* and *gata1a* expression level from 56% to 82%, and from 48% to 84%, respectively (Fig. 1h), injection with 50 pg of mRNA of any *cftr* PDZBD mutant individually failed to rescue the hematopoietic defect in *cftr* mutant—the percentages of embryos with abnormal *scl* and *gata1a* expression level were not significantly changed after the injection (Fig. 1h). Similar results were also observed for definitive hematopoiesis markers, *runx1* and *c-myb*, at 28 hpf and 48 hpf, confirming the importance of PDZBD in the Cftr-regulated hematopoiesis (Appendix Fig. S18).

### PDZBD but not CFTR channel function is required for its interaction with Dvl

G551D is a well-known mutation causing gating defect in CFTR channel function^36,37^. To address whether the regulation of Wnt signaling in hematopoiesis by CFTR is independent of its channel gating function, we tested G551D mutant which has an intact PDZBD. CFTR G551D was able to bind to Dvl2 and significantly enhance the expression of Dvl2 and active-β-catenin in HEK293 cells (Fig. 1f, g). Consistently, 50 pg of G551D mRNA recovered the expression of *scl*/*gata1a* (from 56% to 76%, and from 485 to 64%, respectively) and *runx1/c-myb* (from 42% to 64%, and from 44% to 68%, respectively) in *cftr* mutant embryos (Fig. 1h and Appendix Fig. S18). Collectively, the PDZBD of Cftr, but not its channel function, is essential for maintaining a stable level of Dvl2 protein and Wnt signaling for hematopoiesis.

### Cftr deficiency results in accelerated Dpr1-induced lysosomal degradation of Dvl2

**Fig. 4:**
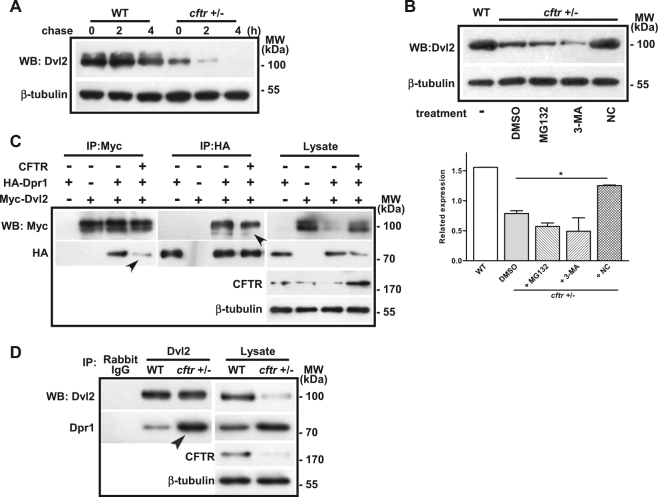
Cftr prevents Dvl2 degradation through DPR1-induced lysosomal pathway. **a** Zebrafish embryos at 5 hpf were CHX chased at the indicated time (in hours). Dvl2 shows degradation pattern in *cftr* mutant zebrafish embryos. The protein levels were analyzed by western blot. **b** Lysosomal inhibitor (NC), but not proteasome inhibitor (MG132) or autophagy inhibitor (3-MA) recovers Dvl2 expression in cftr mutant zebrafish embryos. **c** Co-IP/western blot analysis in HEK293 cells shows that Dvl2 interacts with DPR1, and CFTR overexpression decreases the protein–protein interaction between Dvl2 and DPR1 (marked by arrowheads). **d** Co-IP/western blot analysis in zebrafish embryos shows that endogenous Dvl2 interacts with DPR1, and *cftr* mutants increases the protein–protein interaction between Dvl2 and DPR1 (marked by arrowheads)

The stability of Dvl is regulated by either proteasomal, or lysosomal, or autophagic degradation pathway^21,38^. Our CHX-chase assays using zebrafish embryos demonstrated Dvl2 degradation in *cftr* mutant (Fig. 4a) and morphant (Appendix Fig. S19A) embryos. Then, we used specific inhibitors for these degradation pathways to identify the mechanism for Dvl degradation in *cftr* mutant and morphants. We found that the reduction of Dvl2 in *cftr* mutant (Fig. 4b) and morphants (Appendix Fig. S19B) could be rescued by lysosome inhibitor NH4Cl (NC), but not proteasome inhibitor MG132 or autophagy inhibitor 3-methyladenine (3-MA). These results suggest that deficiency of Cftr results in lysosomal degradation of Dvl2.

We next explored the mechanism by which Cftr prevents lysosomal degradation of Dvl. Since Dpr1 by interacting with Dvl promotes Dvl degradation in lysosomes^24^, we asked whether Cftr may impart its protective effect by attenuating Dpr1-Dvl interaction. Indeed, Co-IP results revealed that exogenous Cftr abrogated the interaction of Dvl2 with DPR1 in HEK293 cells (Fig. 4c). Consistently, we detected increased Dpr1 protein level bound by Dvl2 in *cftr* mutant embryos (Fig. 4d). Taken together, these data suggest that Cftr interferes with the interaction between Dpr1 and Dvl, thus preventing Dpr1-induced Dvl degradation through lysosomal pathway.

### CFTR correlates with DVL, β-CATENIN, and hematopoietic factors in human blood system

**Fig. 5:**
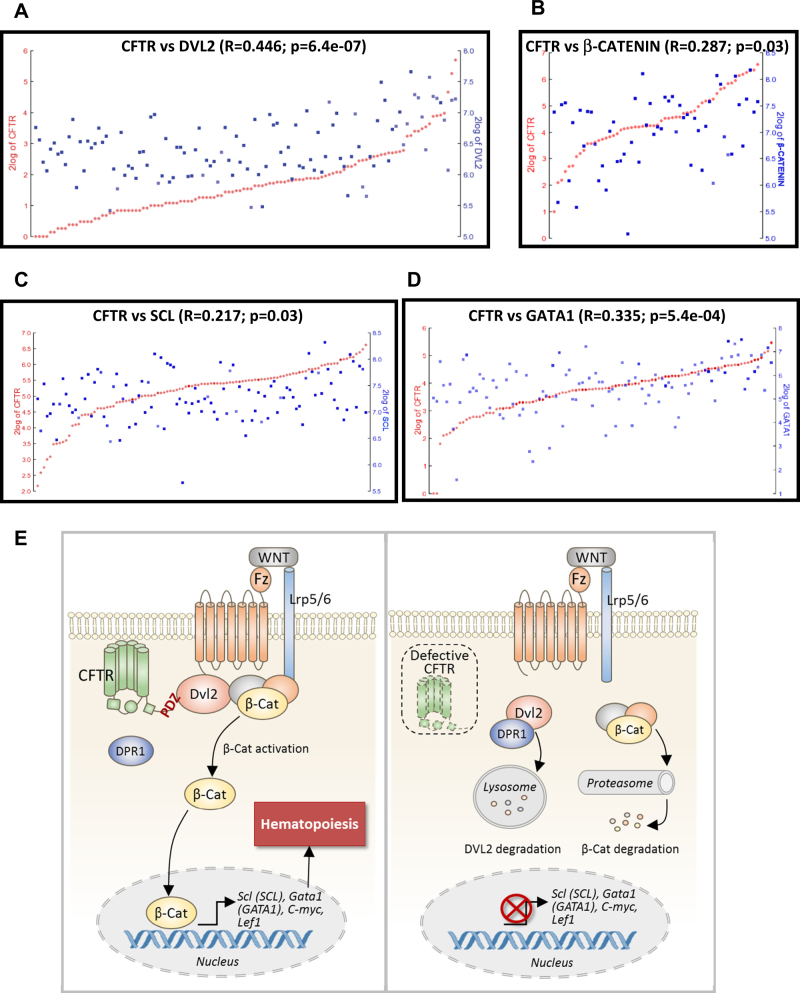
CFTR correlates with DVL2 and key hematopoietic factors in human blood system. R2: Genomics Analysis and Visualization Platform (http://r2.amc.nl) shows that CFTR expression levels positively correlate with DVL2 in human normal leukocytes (**a**), β-CATENIN in human bone marrows (**b**), SCL (**c**), and GATA1 (**d**) in mixed human blood. **e** Model for regulation of Wnt signaling by Cftr in hematopoiesis. Left panel: Cftr (CFTR) binds to Dvl2 (DVL2) through PDZBD and prevents Dvl2(DVL2) from Dpr1-induced lysosomal degradation leading to the activation of Wnt signaling and transcription of hematopoietic target genes in zebrafish and human (in capital). Right panel: defective Cftr (CFTR) results in accelerated Dpr1-induced Dvl2(DVL2) degradation, and thus β-catenin (β-CATENIN) complex degradation, leading to inactivation of Wnt signaling and impaired hematopoiesis. Fz frizzled class receptors, β-cat β-catenin

Since Wnt signaling and hematopoietic factors are evolutionally conserved in vertebrates, the currently demonstrated CFTR involvement in Wnt-dependent hematopoiesis in zebrafish prompted us to examine whether the expression levels of CFTR are correlated with Wnt signaling and related hematopoietic factors in humans. By searching the database R2: Genomics Analysis and Visualization Platform (http://r2.amc.nl), we found that CFTR is positively correlated with DVL2 (*n* = 114, Fig. 5a), β-CATENIN (*n* = 56 Fig. 5b), as well as hematopoietic factors, SCL (*n* = 105, Fig. 5c) and GATA1 (*n* = 103, Fig. 5d) in human blood system, confirming an important role of CFTR in Wnt-dependent hematopoiesis in humans.

## Discussion

While CFTR has been implicated in the organogenesis of the lung^39^ and gut/intestine^40^ with its potential role suggested in regulating Wnt signaling in these developmental contexts, detailed mechanisms have not been elucidated. A recent study using novel deep proteomic analysis has identified a close association of CFTR with the components of Wnt/β-catenin pathway among 638 individual high-confidence CFTR interactors^41^. The present study has provided the first evidence for the involvement of CFTR in the regulation of Wnt-dependent hematopoiesis through its interaction with and regulation of the degradation process of a key adaptor protein of Wnt signaling, Dvl (Fig. 5e).

During primitive hematopoiesis, Wnt cooperates with BMP signaling to promote blood fate through activation of the Cdx-Hox-Scl/Gata1a genetic pathway in embryonic stem cells and zebrafish embryo^19,20^. During definitive hematopoiesis, Wnt signaling is involved in Prostaglandin E2-regulated pathway to drive specification of HSCs^42^. Although most studies have found a positive role for Wnt pathway in HSCs during development^26^, the importance of Wnt signals in normal hematopoiesis is less conclusive^43,44^. The present study demonstrates a crucial role of Wnt signaling in primitive and definitive hematopoiesis, and in addition, we illustrate that the activity of Wnt pathway in this developmental context is regulated by CFTR, a heretofore unknown regulator of hematopoiesis. Furthermore, our findings may provide a tangible etiology for the severe anemia seen in some CF patients^17^, which may be due to a compromised hematopoiesis caused by defective CFTR-dependent Wnt signaling.

Of note, a previous study by Navis et al.^32^ has detected not only the absence of KV but also a defect in the ventral lateral region of the embryo, where hematopoietic progenitor cells emerge, in 10-somite stage *cftr* mutant. This is consistent with the hematopoietic defect evidenced by the reduction of hematopoietic markers observed in this study.

However, despite a remarkable effect on hematopoiesis observed in *cftr* mutants, from both the current and previous studies^32^, the effects of *cftr* mutation/knockdown on the Wnt signaling target genes are relatively mild (Fig. 3b–d). Interestingly, Luis et al.^45^ also reported that canonical Wnt signaling regulates hematopoiesis in a dosage-dependent fashion. They found that only a mild level of activation of Wnt signaling was able to enhance HSC function, whereas, intermediate and high level of activation of Wnt signaling impaired HSC self-renewal or differentiation. However, using Wnt3a−/− mice, the same group demonstrated that Wnt3a deficiency leads to a depletion of the HSC pool, similar to that found with intermediate and high level of Wnt activation^46^. Our finding of a relatively mild effect on Wnt signaling target genes in cftr mutation/knockdown zebrafish (Fig. 3c–e) is consistent with the previous findings^45,46^ and supports the notion that hematopoiesis is regulated by a delicate balance of Wnt signaling levels. Therefore, the variation in Wnt signaling levels in different studies might account for the apparent contradictory findings in mouse HSCs^43,44^.

Although CFTR has been shown to interact and regulate a number of PDZ-containing proteins, including several ion channels^13^ and transporters^47,48^, as well as adaptor proteins that interact with other signaling proteins^49^, it remains an open question whether CFTR channel activity is required for its regulatory role. Most studies used CFTR channel blockers, such as CFTRinh-172, and observed a reversed, reduced, or impaired regulatory effect of CFTR, and interpreted these results as an indication that CFTR channel activity is required for the regulatory action of CFTR. However, these channel blockers have non-specific effects^50^, making the results inconclusive. In the present study, we show that mutations in PDZBD disrupt Cftr-Dvl interaction and fail to support Wnt signaling in zebrafish; however, PDZBD-intact G551D mutation in NBD1, which has been shown to produce CFTR chloride channel defect^36,37^, retains the ability of Cftr to interact with Dvl and rescues Wnt signaling in *cftr* mutant embryos. These results provide compelling evidence for a regulatory role of CFTR that is dependent on its PDZ-binding activity but independent of its channel function. This is consistent with the finding from a previous study of the regulatory effect of CFTR on chemokine RANTES expression in the airways^51^. The authors of this earlier study also noted that the ability of CFTR to restore RANTES expression is not entirely dependent on the amount of CFTR inserted into the plasma membrane. In the present study, we also show that most of the CFTR–Dvl interaction takes place in the cytoplasm, but not at the plasma membrane (Fig. 3c, g), further indicating that this interaction is independent of CFTR channel activity, a function that requires the insertion of CFTR into the plasma membrane.

Our study also provides the first evidence that CFTR can interfere with DPR1–Dvl interaction thereby preventing DPR1-induced lysosomal Dvl degradation. We propose that CFTR competes with DPR1 for binding to Dvl and stabilizes Dvl to maintain a normal level of Wnt signaling required for hematopoiesis (Fig. 4c, d).

The genomic analysis of human blood system database in the present study has also lent strong support for an important role of CFTR in regulating Wnt-dependent hematopoiesis in humans, as demonstrated in the zebrafish model. Not only is CFTR positively correlated with DVL2, but also β-CATENIN as well as the key hematopoietic factors, SCL and GATA, downstream of the Wnt signaling in human blood system (Fig. 5a–d), consistent with an evolutionally conserved role in CFTR in Wnt-regulated hematopoiesis. Given that Wnt signaling plays an important role in hematopoiesis, the presently demonstrated conserved role of CFTR in regulating Wnt signaling suggests its possible involvement in the pathogenesis of different forms of hematopoietic disorders/diseases in humans apart from anemia in CF. Therefore, CFTR may be a potential target for diagnosis and treatment of related hematopoietic diseases.

## Materials and methods

### TALEN-mediated CFTR mutagenesis in zebrafish

The TALENs plasmid pair was kindly provided by Dr. Michel Bagnat (Duke University), and the mutagenesis was performed as Navis et al. described^32^. Differently, we used T7 Endonuclease I assays^52^ (New England Biolabs) instead of EcoRV digestion to identify the mutants. The *cftr* heterozygous mutant embryos were obtained by mating *cftr* heterozygous mutant male fishes with wild-type female fishes.

### Zebrafish embryo manipulation, histology, and in situ hybridization

WISH was carried out as previously described in Thisse et al.^53^ and Sun et al.^54^. After lineage by appropriate restriction enzymes, antisense RNAs for in situ hybridization were synthesized using DIG RNA Labeling Kit (SP6/T7) (Roche) and purified by MEGAclear (Ambion). Two non-overlapping probes, targeting to the sequence 35–1027 bp and 3400–4562 bp, were designed for *cftr* in situ hybridization detection.

Immunofluorescence in zebrafish embryos was performed as previously described (Jia et al.^55^ and Brend et al.^56^) with modifications. Embryos were fixed in fresh 2% paraformaldehyde overnight, permeabilized in 100% methanol at −20 ℃ for at least 1 h. The embryos were bathed in 1 mM EDTA (pH 8.0) at 94–100 ℃ for 10 min and cooled down to room temperature, then incubated in block solution (PBS plus 0.5% Triton X-100 and 1% BSA) for 1 h at room temperature. Embryos were then incubated with a primary antibody at 4 °C overnight, followed by incubation with secondary antibodies (Alexa Fluor® 488 and 594 IgG from Life Technologies Corporation) at room temperature for 1 h. The nuclei were stained by DAPI. The embryos were photographed with Olympus_FV1000 laser scanning confocal microscope.

For immunofluorescence in cells, adherent cells grown on coverslips. All cells were fixed with fresh 4% formaldehyde for 10 min at room temperature, then permeabilized by 0.3% Triton X-100 in PBS for 15 min and blocked with 1% BSA and 0.3% Triton X-100 in PBS buffer. The cells were then probed with primary antibodies, followed by Alexa Fluor 488/546/594 secondary antibodies (Life Technologies Corporation). Fluorescence images were acquired with Olympus_FV1000 laser scanning confocal microscope.

O-dianisidine staining was performed as described by Yue et al.^27^.

Other Materials and Methods are given as Supplementary Information.

## Electronic supplementary material

Supplement

Supplement Figures
