# Supplementary material for: CFTR mutation enhances Dishevelled degradation and results in impairment of Wnt-dependent hematopoiesis
Source: Cell Death Dis. 2018 Feb 15;9(3):275. doi: 10.1038/s41419-018-0311-9 (PMC5833403; doi:10.1038/s41419-018-0311-9)
Supplement: Supplementary file 1 — Supplement [file 41419_2018_311_MOESM1_ESM.docx]

**Appendix - CFTR mutation enhances Dishevelled degradation and results in impairment of Wnt-dependent hematopoiesis**

Sun H, Wang Y, Zhang J, Chen Y, Liu Y, Lin Z, Liu M, Sheng K, Liao H, Tsang KS, Zhang X, Jiang X, Xu W, Mao M, Chan HC

**Table of Contents**

**Appendix Supplementary Materials and Methods**

**Appendix Supplementary Discussion**

**Appendix Table S1**

**Appendix Figure S1.** **Alignment of human CFTR and zebrafish Cftr protein sequence.**

**Appendix Figure S2.** **Spatiotemporal expression pattern of *cftr* transcript in zebrafish embryos.**

**Appendix Figure S3.** ***cftr* mutant zebrafish generated by TALEN.**

**Appendix Figure S4.** **Lateral mesoderm marker *draculin* expression showing insignificant change in *cftr* mutant and morphants.**

**Appendix Figure S5.** **Vascular endothelium marker *flk1* expression showing insignificant change in *cftr* mutant and morphants.**

**Appendix Figure S6.** **The expression of definitive hematopoietic marker genes decreases in *cftr* mutant and morphants at 3 dpf.**

**Appendix Figure S7.** **Specificity of *cftr* morpholinos on hematopoiesis during zebrafish early embryogenesis.**

**Appendix Figure S8. Effectiveness of *cftr* translation-blocker morpholino (*cftr* tMO).**

**Appendix Figure S9.** **Effectiveness of the splice-inhibiting morpholino (*cftr* sMO).**

**Appendix Figure S10. Hematopoietic marker genes expression decreases in *cftr* morphants.**

**Appendix Figure S11.** **Overexpression of Cftr enhances hematopoietic marker genes expression in zebrafish.**

**Appendix Figure S12.** **Decreased expression of Wnt target gene *cdx4* and *hoxa9a* in *cftr* morphants.**

**Appendix Figure S13.** **Reduced Dvl and nuclear β-catenin expression in *cftr* morphants.**

**Appendix Figure S14.** ***dvl2* mRNA rescues the hematopoietic defect in *cftr* morphants.**

**Appendix Figure S15. Western blot and quantitative RT-PCR show that *dvl2* mRNA injection recovered the expression of active-β-catenin (A) and Wnt target genes, *c-myc* and *lef1* (B), in *cftr* mutant embryos.**

**Appendix Figure S16. Effects of *dvl2* mRNA in rescuing definitive hematopoietic defects in cftr mutants.**

**Appendix Figure S17. Co-immunoprecipitation (Co-IP) of endogenous Dvl2 and Cftr showing that Dvl2 is interacted with Cftr in mouse bone marrow.**

**Appendix Figure S18. Effects of different *cftr* mutants mRNA in rescuing definitive hematopoietic defects in *cftr* mutant zebrafish embryos.**

**Appendix Figure S19. Dvl2 degradation through lysosomal pathway in *cftr* morphants.**

**Appendix References**

**Appendix Supplementary Materials and Methods**

***Reagents and antibodies.***

3-methyladenine (3-MA, 5142-23-4), Ammonium chloride (NH_4_Cl, NC, 254134) and DAPI (D9542) were from Sigma. MG132 (474790) and PureProteome Protein G Magnetic Beads (LSKMAGG10) were from Millipore. Normal rabbit IgG (sc-2027) and normal mouse IgG (sc-2025) were from Santa Cruz. Cycloheximide (CHX, S1560) was from Beyotime Biotechnology. The following antibodies were used: anti-CFTR (Abcam, ab2784), anti-c-Myc (Santa Cruz, sc-40), anti-HA (Santa Cruz, sc-805), anti-Dvl2 (Rabbit polyclonal antibody, ProteinTech, 12037-1-AP), anti-Dvl2 (Goat polyclonal antibody, Santa Cruz, sc-7399), anti-Dvl3 (ProteinTech, 13444-1-AP), anti-β-catenin ([Cell Signaling Technology](http://product.bio1000.com/brand/info/70.htm), 9562), anti-active-β-catenin (Millipore, 05-665), anti-β-tubulin (Epitomics, 1879-1 and Zen Bioscience, 200608), anti-Histone H2B (Abcam, ab52985), anti-Dapper1 (DACT1, Invitrogen, PA5-23216).

***Ethics Statement.***

All experiments in this study were in accordance with the “Guide for the Care and Use of Laboratory Animals” (Eighth Edition, 2011. ILARCLS, National Research Council, Washington, D.C.) and were approved by the Animal Care and Use Committee of West China Second University Hospital, Sichuan University (Approval ID: HXDEYY20101105).

***Zebrafish Embryos.***

WT embryos from AB strain were used. Embryos of *flk1* gene green fluorescence were generated by transgenenic fish line *Tg(flk1:GFP)*. Embryos were obtained by natural mating and cultured in embryo medium^1^. Staging of the embryos was carried out according to Kimmel *et al*^2^.

***Morpholino oligonucleotides, in vitro synthesis of RNA and microinjection.***

Three *cftr* morpholino antisense oligonucleotides, a translation-blocker (*cftr* tMO, 5’-CATCCTCCACAGGTGATCTCTGCAT-3’), a splice-inhibitor (*cftr* sMO, 5’-AAGAGCGTTTAGACTCACTTCAATC-3’) and 5-mis-pair control morpholino (*cftr* cMO, 5’-CATCCTCCAGACGTCATCTGTCCAT-3’) were designed and synthesized by Gene-tools, Philomath, OR. Capped mRNAs were synthesized using mMESSAGE mMACHINE^®^ Kit (Ambion); Morpholinos or synthetic capped mRNAs were injected into single-cell embryos. Injection dose was an estimated amount received by a single embryo.

***Constructs.***

Zebrafish *cftr* full length mRNA sequence was from openbiosystems (Catalog Number EDR5649-100965960). The coding region was amplified by primer pair *cftr*-cds (Appendix Table S1) and cloned into pcDNA3.1+ vector (Invitrogen) for capped mRNA synthesis and transfection; Myc/HA-tag coding sequence was added to upstream of *cftr* and *dvl*1-3 cDNA respectively, and fused sequences were also cloned into pcDNA3.1+ for capped mRNAs synthesis and transfection; The N terminal 500 bp of *cftr* coding region was cloned into pEGFP-N1 for expression of *cftr*-N500-GFP fusion protein to test effectiveness of *cftr* tMO; The PDZ-binding/PDZ domain deletion and point mutation expression plasmids of *cftr* (point substitutions in the C-terminus are underlined:HA-CFTR-DTRA, HA-CFTR-DARL) and *dvl2* were constructed using KOD-Plus-Mutagenesis Kit (TOYOBO); Fragments of hematopoietic marker genes were cloned into pEASY-T3 (Transgen) for antisense RNA probe synthesis; Coding regions of wnt3a were also cloned into pcDNA3.1+ for mRNAs synthesis; TopFlash-luciferase and HA-DPR1 plasmid were kindly provided by Prof Chen Ye-Guang (Tsinghua University).

***Subcellular fractionation,*** ***Western blotting and Immunoprecipitation.***

Cytoplasmic and nuclear extracts were prepared using the hypotonic buffer (20 mM HEPES, pH 8.0, 10 mM KCl, 1 mM MgCl2, 0.1% Triton X-100, and 20% glycerol) and insoluble buffer (20 mM Tris, pH 8.0, 150 mM NaCl, 1% SDS, 1% NP-40), respectively^3^. Whole-cell extracts were prepared by lysing cells in Non-denaturing lysis buffer (20 mM Tris HCl pH 8, 137 mM NaCl, 10% glycerol, 1% NP-40, 2 mM EDTA). All the lysis buffers were supplemented with protease inhibitor cocktail (Cat. No. 04 693 132 001, Roche).

For protein detection in zebrafish embryo, embryos were deyolked firstly. In detailed, embryos were collected in glass homogenizer and washed by deyolking buffer three times; the embryo chorions and yolk were disrupted by glass homogenizer in deyolking buffer (55 mM NaCl, 1.8 mM KCl, 1.25 mM NaHCO3)^4^; the disrupted embryos were transferred to 15 ml tube and vortexed 10 sec to separate cells from yolk sac protein; cells were pelleted at 300 g for 3 min with horizontal rotor and the supernatant discarded; proper volume of Non-denaturing lysis buffer was added and continued with subsequent performance.

For immunoprecipitation, embryo or cell extract was precleared first by PureProteome Protein G Magnetic Beads and mixed with 2 μg antibody at 4℃ overnight. Then, 50 μl PureProteome Protein G Magnetic Beads were used as the product guideline described. Finally, the beads were directly incubated at 37℃ in 1×SDS loading buffer for 15 min. Bone marrows were collected from mouse thigh. After washing by PBS buffer, protein was extracted by Non-denaturing lysis buffer and immunoprecipitation was performed as above.

***Cell culture, siRNA, Transfection and Luciferase Reporter Assays***

HEK293 cells were grown in DMEM plus 10% FBS. DNA transfection into HEK293 cells was performed using jetPRIME (Polyplus). Plasmid pcDNA3.1+ inserted with GFP coding region was used as transfection control. INTERFERin (Polyplus) was used for siRNA transfection (final concentration, 50 nM). Human *CFTR* siRNA pair (5’ CGCGAUUUAUCUAGGCAUA dTdT 3’ and 3’ dTdT GCGCUAAAUAGAUCCGUAU 5’) and common negative control siRNA were designed and synthesized by Ribobio (Guangzhou, China). For luciferase Reporter Assays, HEK293 cells were transfected with various plasmids. One day after transfection, the culture medium was changed and *CFTR* siRNA was transfected. Cells were harvested at two days after first transfection and luciferase activities were measured according to Dual-Glo® Luciferase Assay System (Promega). Reporter activity was normalized to the control Renilla. Experiments were repeated in triplicate.

***Quantitative real time RT–PCR (qRT–PCR) analysis.***

Total RNA was prepared with TRIzol (Invitrogen, 15596-018) and cDNA was synthesized from 1 μg of RNA with PrimeScript RT reagent Kit (Takara, DRR037A). qRT–PCR was performed with the SYBR Green detection method with 7500 real-time PCR system (Applied Biosystems). The primers used were shown in Table S1.

***In vitro protein synthesis and binding assay.***

In vitro proteins synthesis was performed using TnT® Quick Coupled in vitro transcription/translation system (Promega) according to manufacturer’s instructions. In vitro cell-free Cftr and Dvl2 protein expression were carried out in two reactions using TnT® system separately. Subsequently, each of the synthesized protein were mixed together in The Non-denaturing lysis buffer and incubated on a rotating platform at 4℃ overnight. In vitro binding assay between Cftr and Dvl2 was detected by Co-IP and Western blotting using anti-HA and anti-c-myc antibody.

***Reagents treatment of zebrafish embryos and protein stability assay.***

At the beginning of gastrula (5 hpf), the embryos (20 embryos in a well of 6-well plate with 2 ml culture water) were treated with DMSO, proteasome inhibitor MG132(10μM), autophagy inhibitor 3-MA (5mM) or lysosomal inhibitor NH4Cl (NC) (2.5mM) for 2h and then subject to western blotting. For protein stability assay, zebrafish embryos at 5 hpf were treated with 10μM CHX, followed by chase of the indicated time period, and western blotting to detect the indicated proteins.

***Grayscale measurement, immunostaining colocalization analysis and statistics.***

Signal strength grayscale of whole-mount in situ hybridization and western blotting assay was measured by software ImageJ*.* Statistical analyses were performed with a Student’s t test. Quantitative data show the mean+SD. Statistical significance is defined as P<0.05(*), P<0.01(**), P<0.001(***). Immunostaining colocalization was analyzed by Olympus_FV1000 of confocal microscope. Parameters, “Pearson’s coefficient” (Pearson’s coeff) and “Overlap” were key index, ranging from -1 to 1, with 1 indicating 100% colocalization.

**Appendix Supplementary Discussion**

The present finding is also consistent with the recently discovered role of CFTR in mesoendoderm differentiation of mESCs^5^ since hematopoietic progenitors emerge from the mesoderm. However, CFTR interacts with β-catenin, but not Dvl, in mESCs^5^. Interestingly, CFTR has also been found to interact with Dvl, but not β-catenin, in kidney epithelial cell lines^6^. It appears that CFTR may interact with different components of the Wnt/β-catenin signaling pathway, i.e. β-catenin or Dvl, in different cellular contexts, suggesting tissue-specific role of CFTR. The exact molecular mechanism governing differential interaction of CFTR with different proteins in different cell types remains to be elucidated.

Wnt signaling controls a variety of developmental and homeostatic events, although the underlying mechanisms remain far from clear. As a key component of Wnt signaling, Dvl protein relays Wnt signals from receptors to downstream effectors. In the canonical Wnt pathway that depends on the nuclear translocation of β-catenin, Dvl is recruited by the receptor Frizzled and prevents the constitutive destruction of cytosolic β-catenin. Additionally, in the non-canonical Wnt pathways such as Wnt-Frizzled/PCP (planar cell polarity) signaling, Dvl signals via the Daam1-RhoA axis and the Rac1 axis. Furthermore, Dvl plays important roles in Wnt-GSK3β-microtubule signaling, Wnt-calcium signaling, Wnt-RYK signaling, Wnt-atypical PKC signaling, etc. Dvl also functions to mediate receptor endocytosis. Given its hub position and association with more than ﬁfty binding partners, it is not surprising that Dvl mediates complex and versatile signal transduction^7^. On the other side, Pankow *et al.* used novel deep proteomic analysis to identify a close association of CFTR with components of Wnt/β-catenin pathway among 638 individual high-confidence CFTR interactors^8^. Taken together, identiﬁcation and characterization of the CFTR/Wnt-involved protein–protein interactions will undoubtedly increase our comprehension in the enigmatic features of CFTR/Wnt in tissue-specific biological process.

**Appendix Table**

**Appendix Table S1:** Oligonucleotide primers and probes used in the study

|  | | |
| --- | --- | --- |
| **Primer** | **Forward 5’-3’** | **Reverse 5’-3’** |
| *cftr*-cds | CGGAATTCGATGCAGAGATCACCTGTGGAGGATG | GCTCTAGATCAGAGGCGAGTGTCCTGGATGTTG |
| *dvl2*-cds | CCCAAGCTTGGGTTGAGAGACATGGCGGAGACC | CGGAATTCCATCCTTCACTCGGGTACATCCTTC |
| *cftr*-probe1 | GCGTTGAGCTGATACCCGTAACCCG | GTACGCCGAGCTGTAGAAGTAGCGC |
| *cftr*-probe2 | CACTCTCCGATGGTTCCTCTTCCGC | TTTGGAACGCTCAGAGGCGAGTGTC |
| Ex7f/9R | GAC AGG ATG AGG TGA AGC | CCC ATT AGC TTT GTT CTC |
| *scl*-probe | TCTGGGATCGCGCCGAAGGATGATG | CAGTCACCGCTGGGCATTTCCGTCC |
| *fli1a*-probe | ATGGACGGAACTATTAAGGAGGCGC | CCTTCTCCATCTTCGAGTGCAGTTC |
| *lmo2*-probe | GTGCGGCAGATGGTCTTTCGGAACC | CTGCAACCCAACACTGGGAAACCTG |
| *gata2a*-probe | GCGGACACTTGAGATGGAGGTTGCG | GCTGGGTTGTTCAGTGTGGTTCGGC |
| *runx1*-probe | GGCACGAGCCGAAACTCACGGAGAC | CGGCGTGCTATTGGCTGTCAGTATG |
| *pu.1*-probe | GCACGAGTACAGAATGGAGGGGTAC | CTCCCGTCTTTCCGTAGTTTCTCAG |
| *gata1a*-probe | GCAGATGGAGAACTCCTCTGAGCCT | GGTCCCGTGGATGTTTCCTTCACAC |
| *hbbe3*-probe | ATGGTTGTGTGGACAGCTGAGGAGC | CTAAAGATATTGCCTTCTGAGGGCT |
| *c-myb*-probe | AGCGGGATGGCGAGGCGGCACAGAC | TCTGTTCCAGCCACCGTAGCCGTTC |
| *draculin*-probe | CACTCCACCCTAAATACAAGCC | GAAATTAATACGACTCACTATAGGGAGACCCTTTCTCATGCAACCTCAAACCC |
| *Wnt3a*-cds | CGGGATCCCGCGTGTGCGTGTCAGTTTAAGCCT | CGGAATTCCTCCCTTGCGTGTTCACATGTGTCT |
| zebrafish *dvl2*-qRT-PCR | AGCTGTAGCTGCAGATGGAC | GGTGATGGGTCCAGGTTTGT |
| zebrafish *dvl3*-qRT-PCR | CCCCGAGACCGAGAGATTTG | AAAGCCAGTCCACCACATCC |
| zebrafish *c-myc*-qRT-PCR | AACACAAGTGTCAAAATGCCG | TTCCAAATGTCCTCGCTGGG |
| zebrafish *lef1*-qRT-PCR | CCGGATGATGGCAAACTCCA | TTCGACGTTCTGGGAATGGG |
| zebrafish *β-actin*-qRT-PCR | ATGAGTCTGGCCCATCCATC | CCTTTGCCAGTTTCCGCATC |

**Appendix Figure legends**

**Appendix Figure S1.** **Alignment of human CFTR and zebrafish Cftr protein sequence.** (A) Complete sequence alignment. (B) Conserved PDZBD (marked by red box) in human CFTR and zebrafish Cftr protein.

**Appendix Figure S2.** **Spatiotemporal expression pattern of *cftr* transcript in zebrafish embryos.** (A) Location of anti-sense RNA probe for detection of zebrafish Cftr using WISH. (B) Detection by Probe 1 at indicated stages. (C) Detection by Probe 2 at indicated stages. Arrows show the expression region of *cftr*. Arrowheads in 70%-epiboly stage show the forerunner cells, which migrate deep into the embryo and organize to form Kupffer's vesicle, consistent with Navis et al^9^. The same expression pattern of *cftr* shown by two non-overlapping probes, demonstrating the specificity and identical spatially restricted expression patterns of *cftr* in zebrafish early embryogenesis^10^. Embryo orientations: 2-cell and Sphere stage, lateral views with the animal pole oriented at the top; 75%-epiboly stage, dorsal view with the animal pole oriented at the top; 5-somite and Prim-5 stage, lateral views with anterior oriented toward the left. The indicated domains: ba, branchial and pharyngeal arches.

**Appendix Figure S3.** ***cftr* mutant zebrafish generated by TALEN.** (A) Genomic sequence alignments of the TALEN-generated alleles. The obtained mutant has two-nucleotide deletion in Exon 6, causing frameshifts leading to premature stop codons at 219 AA. (B) An alignment of the amino acid sequences encoded by *cftr* mutant and wild type *cftr.*

**Appendix Figure S4.** **Lateral mesoderm marker *draculin* expression showing insignificant change in *cftr* mutant and morphants.** 4ng *cftr* cMO or tMO was injected into the embryos, and then hybridized with indicated probes. 4-somite stage embryos are dorsal views with anterior oriented at the top; Shield embryos are top views with dorsal to the right. Arrowheads indicate the expression sites of the marker gene.

**Appendix Figure S5.** **Vascular endothelium marker *flk1* expression showing insignificant change in *cftr* mutant and morphants.** Transgenic fish line *Tg(flk1:GFP)* was used for this assay. 4ng *cftr* cMO or tMO was injected into the embryos, and then photoed by Nikon Eclipse Ti-U fluorescence microscope. All embryos shown are lateral views with anterior oriented at the left. Arrowheads indicate the expression sites of artery.

**Appendix Figure S6.** **The expression of definitive hematopoietic marker genes decreases in *cftr* mutant and morphants at 3 dpf.** Progenitor cells marked by *scl*, erythroid marked by *gata1a* and myeloid marked by *l-plastin.* 4ng *cftr* cMO or tMO was injected into the embryos, and then hybridized with indicated probes. All embryos shown are lateral views with anterior oriented at the left. Arrowheads indicate the expression sites of marker gene.

**Appendix Figure S7.** **Specificity of *cftr* morpholinos on hematopoiesis during zebrafish early embryogenesis.** 4ng *cftr* MOs was coinjected with 50pg *CFTR* mRNA into the embryos, then hybridized with *scl* probe. (*cftr* tMO, translation-blocker morpholino; cMO, 5-mis-pair control morpholino). The ratio and number of calculated embryos are indicated at bottom in each image. According to Judith S. Eisen’s recommendations^11^, a splice-inhibitor morpholino (*cftr* sMO, detail in Appendix Figure S6) was used to identify the specific effects of *cftr* tMO. Embryos shown are dorsal views with anterior oriented at the top at 8-somite stage.

**Appendix Figure S8. Effectiveness of *cftr* translation-blocker morpholino (*cftr* tMO).** Live embryos at the 70%-epiboly stage. (A) non-injection embryos. (B) Embryos injected with 400pg *cftr*-N500-*GFP* DNA showing the expression of Cftr-N500-GFP fusion protein. (C) Embryos coinjected with 400pg *cftr*-N500-*GFP* DNA and 4ng *cftr* tMO showing decreased expression of GFP fusion protein level compared to (B). (D) Embryos coinjected with 400pg *cftr-*N500-*GFP* DNA and 4ng *cftr* cMO showing no significant change in the expression of GFP fusion protein level compared to (B).

**Appendix Figure S9.** **Effectiveness of the splice-inhibiting morpholino (*cftr* sMO).** (A) Parts of genomic structure of zebrafish *cftr* gene. Exons and primers are indicated. Splice site targeted by *cftr* sMO is shown. (B) RT-PCR assay detecting the effectiveness of *cftr* sMO. Injection of 5ng *cftr* sMO produces diminution of the wild type transcript band at the Shield stage (6.5 hpf), suggesting that *cftr* sMO specifically target zygotic transcripts^12^. *cftr* sMO, locating at the exon8-intron8 boundary, was designed by Gene Tools, LLC and would generate an out-of-frame mutation resulting in a loss-of-function phenotype. Injection of *cftr* sMO can produce pre-mRNA sequence with a premature termination codon brought in-frame by the exon8 excision and the pre-mRNA will undergo nonsense-mediated decay^13^.

**Appendix Figure S10. Hematopoietic marker genes expression decreases in *cftr* morphants.** 4ng *cftr* cMO or tMO was injected into the embryos, and then hybridized with indicated probes. (A) WISH for embryos at 8-somite stage (13 hpf). Embryos shown are dorsal views with anterior oriented at the top. (B)WISH for embryos at later stage. Embryos shown are lateral views with anterior to the left. Hemoglobin staining by O-dianisidine at 36 hpf. Embryos shown are ventral views with anterior to the left. Arrowheads indicate the expression sites of each marker gene.

**Appendix Figure S11.** **Overexpression of Cftr enhances hematopoietic marker genes expression in zebrafish.** Embryos injected with *GFP* mRNA or *cftr* mRNA (100pg) were hybridized with *scl* and *gata1a* probes at 8-somite stage.

**Appendix Figure S12.** **Decreased expression of Wnt target gene *cdx4* and *hoxa9a* in *cftr* morphants.** Embryos injected with *cftr* cMO or tMO (4ng) were hybridized with *cdx4* and *hoxa9a* probes at 8-somite stage.

**Appendix Figure S13.** **Reduced Dvl and nuclear β-catenin expression in *cftr* morphants.** Embryos injected with *cftr* cMO or tMO (4ng), and then perform western blotting to show the expression of Dvl2 and nuclear β-catenin. Histone H2B served as nuclear protein loading control and β-tubulin served as total protein loading control.

**Appendix Figure S14.** ***dvl2* mRNA rescues the hematopoietic defect in *cftr* morphants.** Embryos coinjected with *dvl2* mRNA (30pg) and *cftr* cMO or tMO(4ng). Whole-mount in situ hybridization monitoring *scl* and *gata1a* expression at 8-somite stage (13 hpf). Embryos shown are dorsal views with anterior to the top.

**Appendix Figure S15. Western blot and quantitative RT-PCR show that *dvl2* mRNA injection recovered the expression of active-β-catenin (A) and Wnt target genes, *c-myc* and *lef1* (B), in *cftr* mutant embryos.**

**Appendix Figure S16. Effects of *dvl2* mRNA in rescuing definitive hematopoietic defects in cftr mutants.** All embryos shown are lateral views with anterior to the left.

**Appendix Figure S17. Co-immunoprecipitation (Co-IP) of endogenous Dvl2 and Cftr showing that Dvl2 is interacted with Cftr in mouse bone marrow.**

**Appendix Figure S18. Effects of different *cftr* mutants mRNA in rescuing definitive hematopoietic defects in *cftr* mutant zebrafish embryos.** Injection of *cftr* PDZBD mutants, either deletion or point mutation, could not rescue the definitive hematopoietic defect in *cftr* mutant embryos. Injection of G551D rescues the definitive hematopoietic defect in *cftr* mutant embryos. All embryos shown are lateral views with anterior to the left.

**Appendix Figure S19. Dvl2 degradation through lysosomal pathway in *cftr* morphants.** (A) Zebrafish embryos injected with *cftr* cMO or tMO (4ng) at 5 hpf were CHX chased at the indicated time (in hours). Dvl2 shows degradation pattern in *cftr* morphants. (B) Lysosomal inhibitor recovers Dvl2 expression in *cftr* morphants. Embryos injected with *cftr* cMO or tMO (4ng), and then treated with DMSO, MG132 (10μM), 3-MA (5mM) or NC (2.5mM) for 2h. Western blotting showing the Dvl2 expression level.

**Appendix References**

1. Westerfield M. *The Zebrafish Book*. University of Oregon Press: Oregon, 1993.

2. Kimmel CB, Ballard WW, Kimmel SR, Ullmann B, Schilling TF. Stages of embryonic development of the zebrafish. *Dev Dyn* 1995; **203**(3)**:** 253-310.

3. Yan P, Fu J, Qu Z, Li S, Tanaka T, Grusby MJ*, et al.* PDLIM2 suppresses human T-cell leukemia virus type I Tax-mediated tumorigenesis by targeting Tax into the nuclear matrix for proteasomal degradation. *Blood* 2009; **113**(18)**:** 4370-4380.

4. Link V, Shevchenko A, Heisenberg CP. Proteomics of early zebrafish embryos. *BMC Dev Biol* 2006; **6:** 1.

5. Liu Z, Guo J, Wang Y, Weng Z, Huang B, Yu MK*, et al.* CFTR-beta-catenin interaction regulates mouse embryonic stem cell differentiation and embryonic development. *Cell Death Differ* 2017; **24**(1)**:** 98-110.

6. Zhang JT, Wang Y, Chen JJ, Zhang XH, Dong JD, Tsang LL*, et al.* Defective CFTR leads to aberrant beta-catenin activation and kidney fibrosis. *Sci Rep* 2017; **7**(1)**:** 5233.

7. Gao C, Chen YG. Dishevelled: The hub of Wnt signaling. *Cellular signalling* 2010; **22**(5)**:** 717-727.

8. Pankow S, Bamberger C, Calzolari D, Martinez-Bartolome S, Lavallee-Adam M, Balch WE*, et al.* F508 CFTR interactome remodelling promotes rescue of cystic fibrosis. *Nature* 2015; **528**(7583)**:** 510-516.

9. Navis A, Marjoram L, Bagnat M. Cftr controls lumen expansion and function of Kupffer's vesicle in zebrafish. *Development* 2013; **140**(8)**:** 1703-1712.

10. Thisse C, Thisse B. High-resolution in situ hybridization to whole-mount zebrafish embryos. *Nature protocols* 2008; **3**(1)**:** 59-69.

11. Eisen JS, Smith JC. Controlling morpholino experiments: don't stop making antisense. *Development* 2008; **135**(10)**:** 1735-1743.

12. Draper BW, Morcos PA, Kimmel CB. Inhibition of zebrafish fgf8 pre-mRNA splicing with morpholino oligos: a quantifiable method for gene knockdown. *Genesis* 2001; **30**(3)**:** 154-156.

13. Baker KE, Parker R. Nonsense-mediated mRNA decay: terminating erroneous gene expression. *Curr Opin Cell Biol* 2004; **16**(3)**:** 293-299.
