# Supplementary material for: CFTR mutation enhances Dishevelled degradation and results in impairment of Wnt-dependent hematopoiesis
Source: Cell Death Dis. 2018 Feb 15;9(3):275. doi: 10.1038/s41419-018-0311-9 (PMC5833403; doi:10.1038/s41419-018-0311-9)
Supplement: Supplementary file 2 — Supplement Figures [file 41419_2018_311_MOESM2_ESM.pdf]

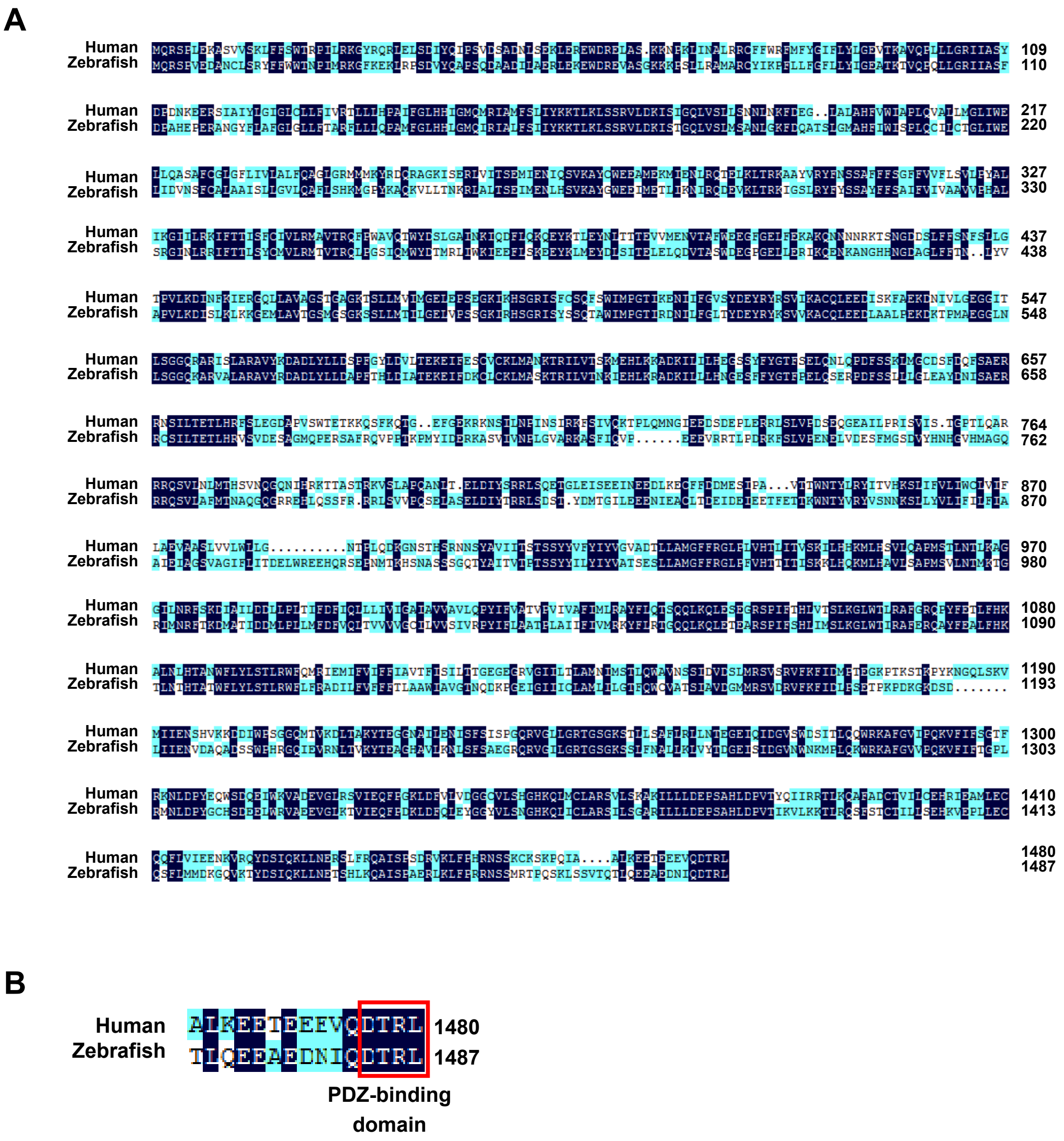

Supplementary figure 1

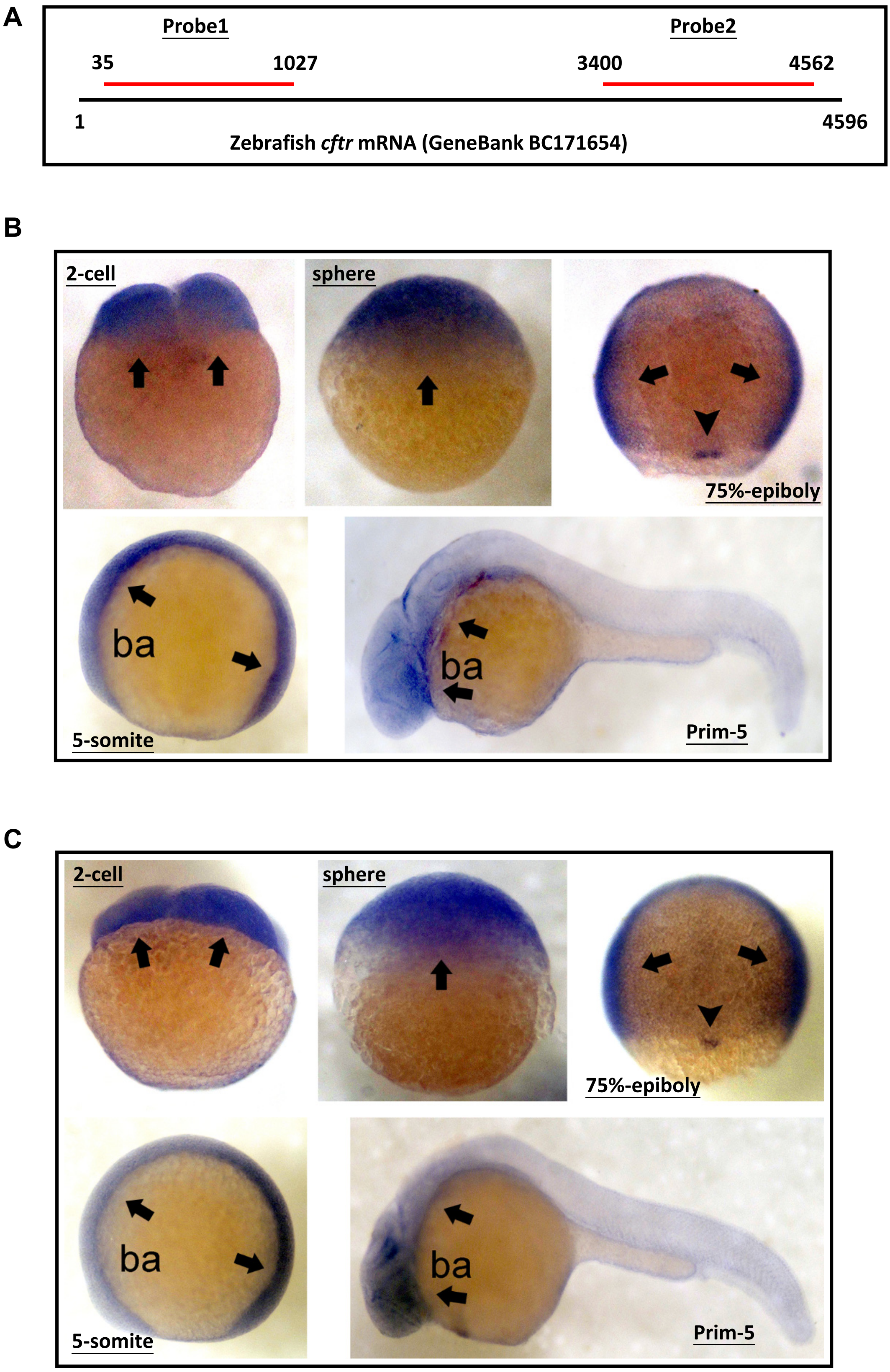

Supplementary figure 2

A

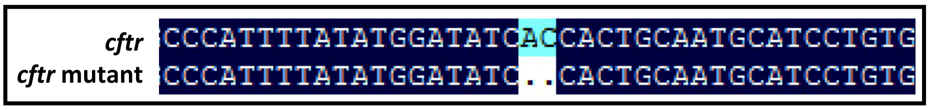

B

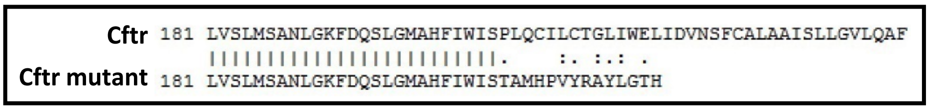

Supplementary figure 3

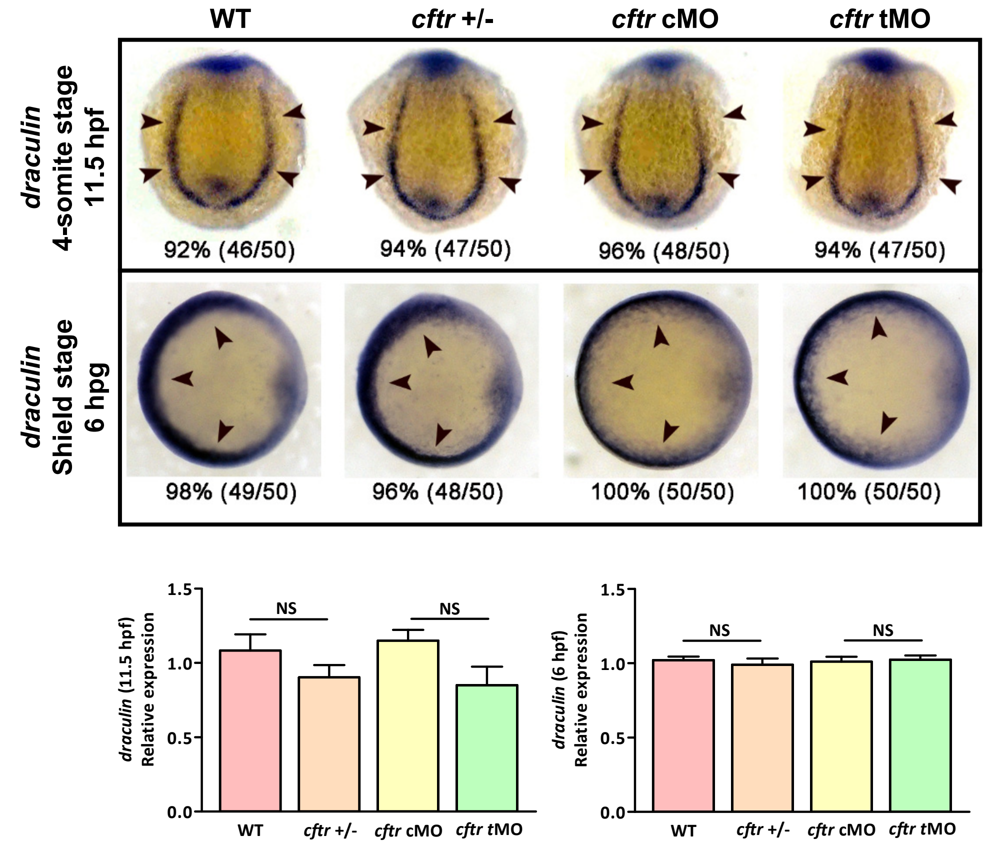

Supplementary figure 4

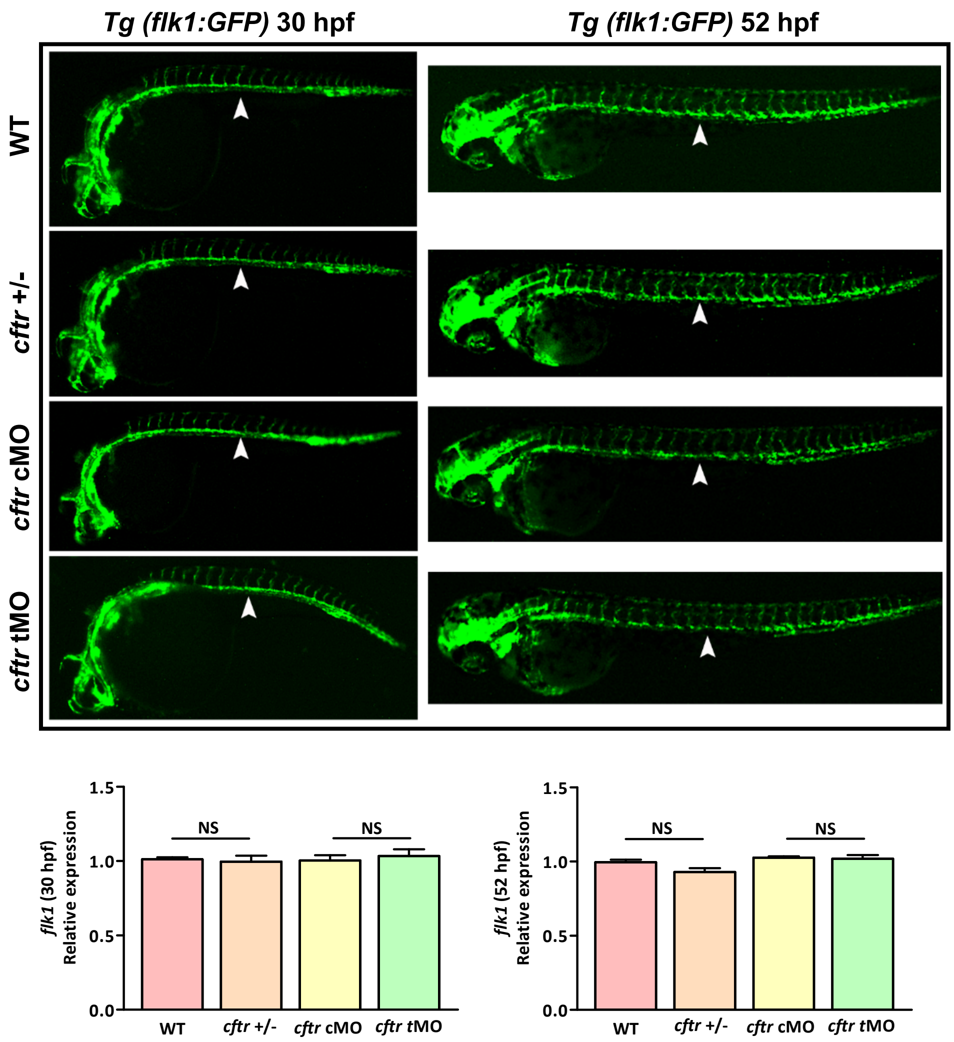

Supplementary figure 5

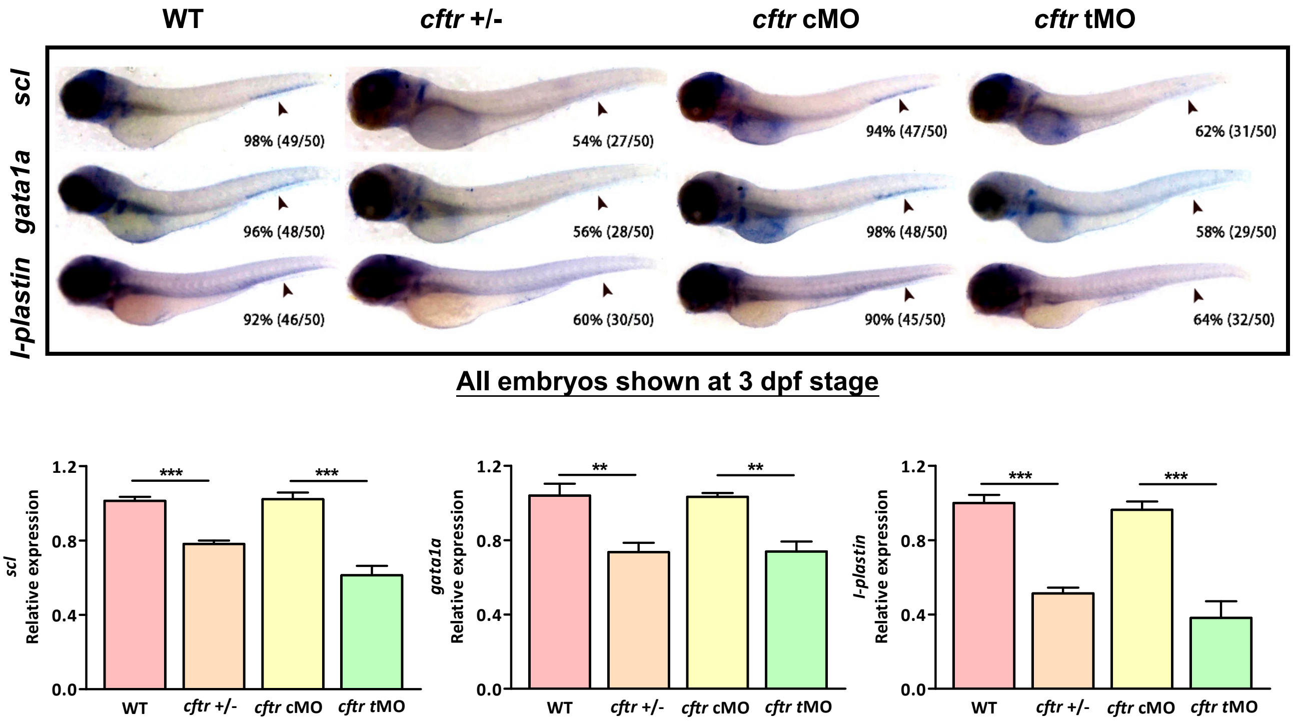

Supplementary figure 6

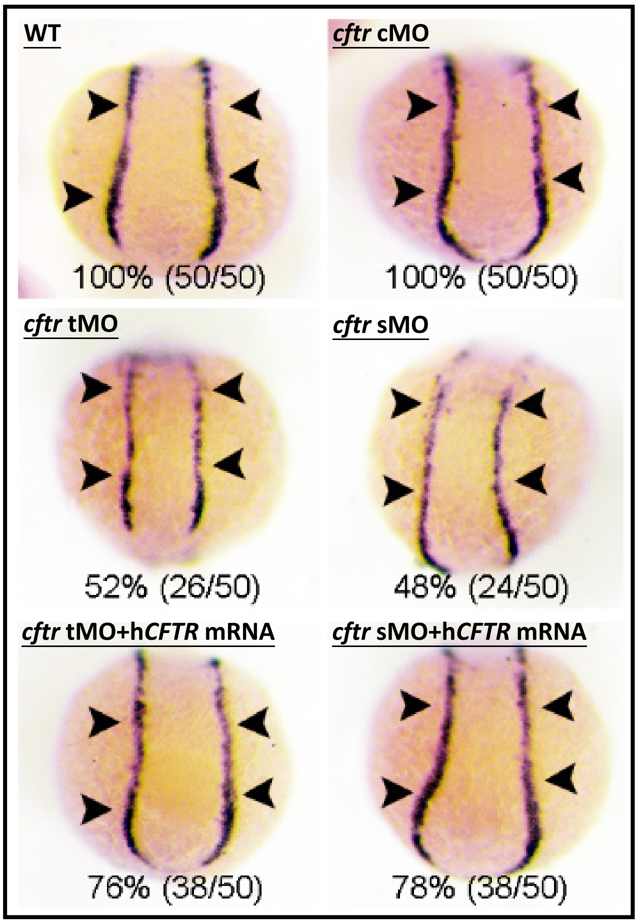

All embryos shown at 8-somite stage (13 hpf)

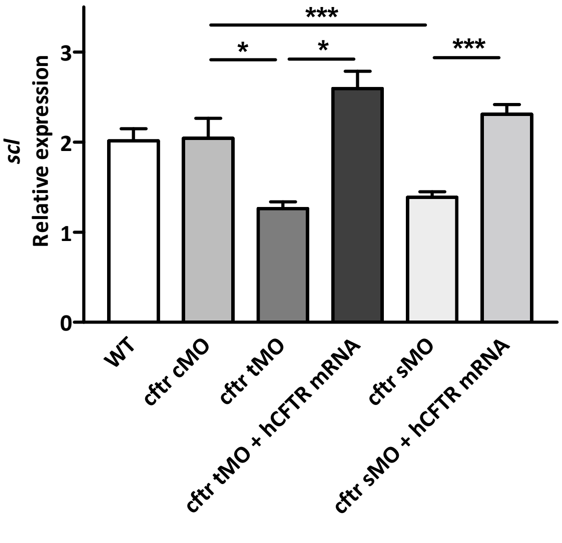

Supplementary figure 7

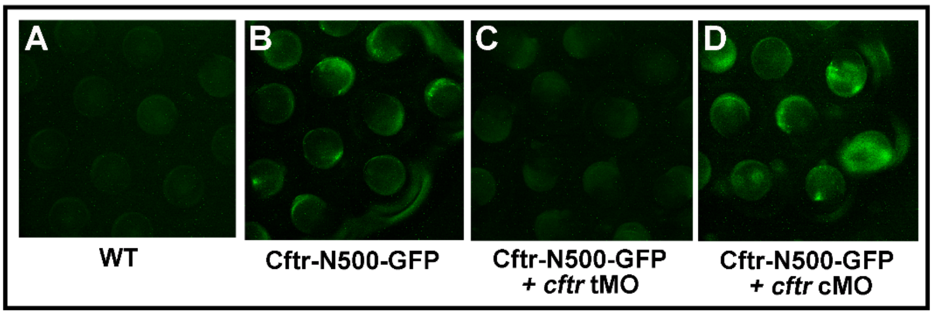

All embryos shown at 70%-epiboly stage (7.5 hpf)

Supplementary figure 8

A

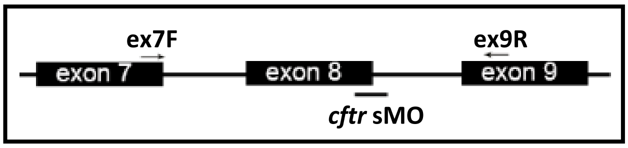

B

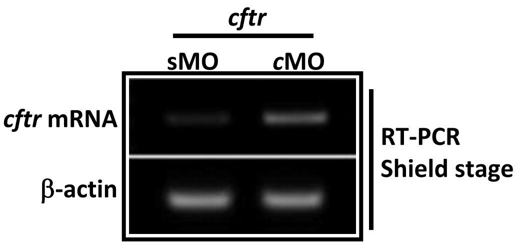

Supplementary figure 9

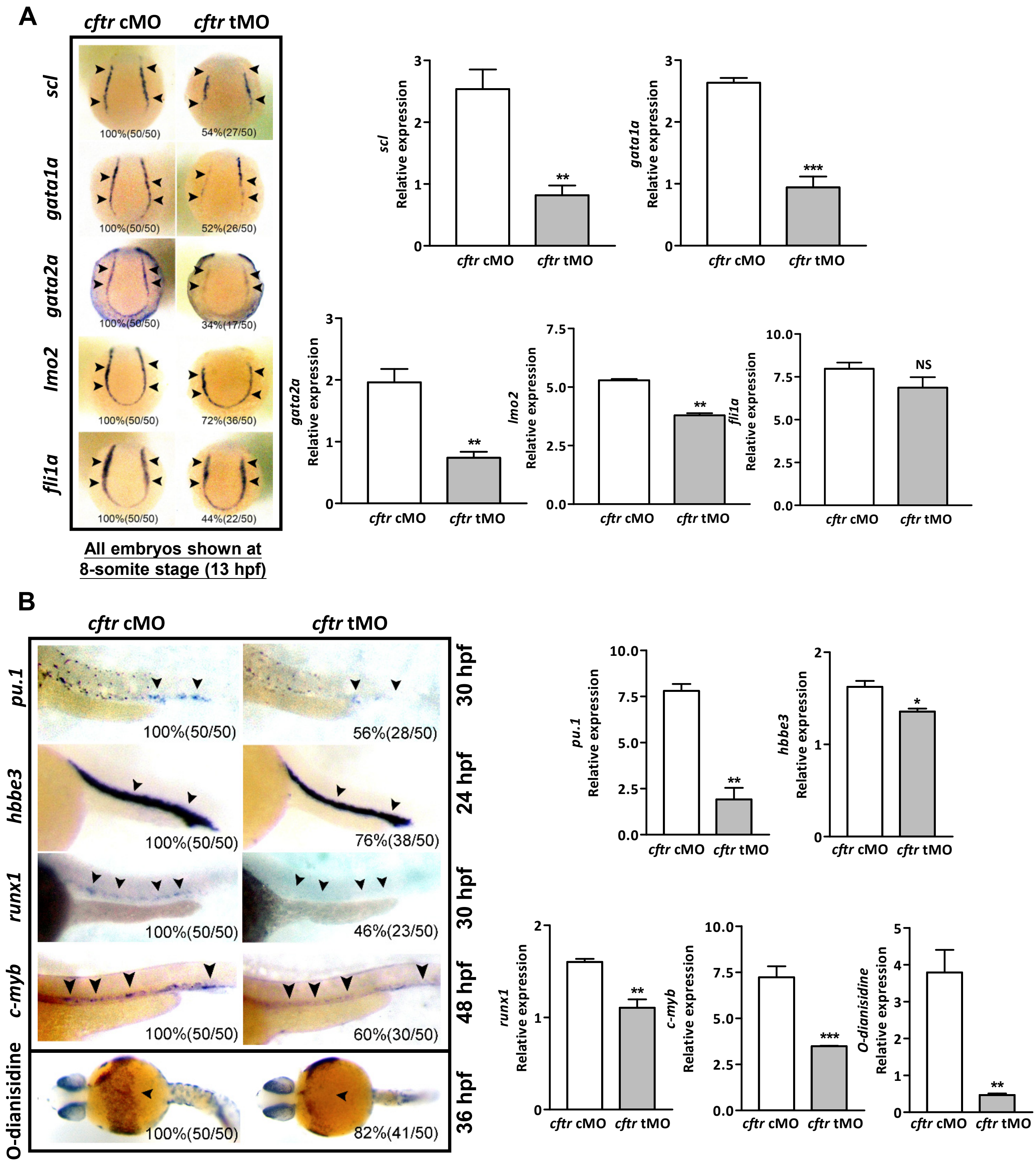

Supplementary figure 10

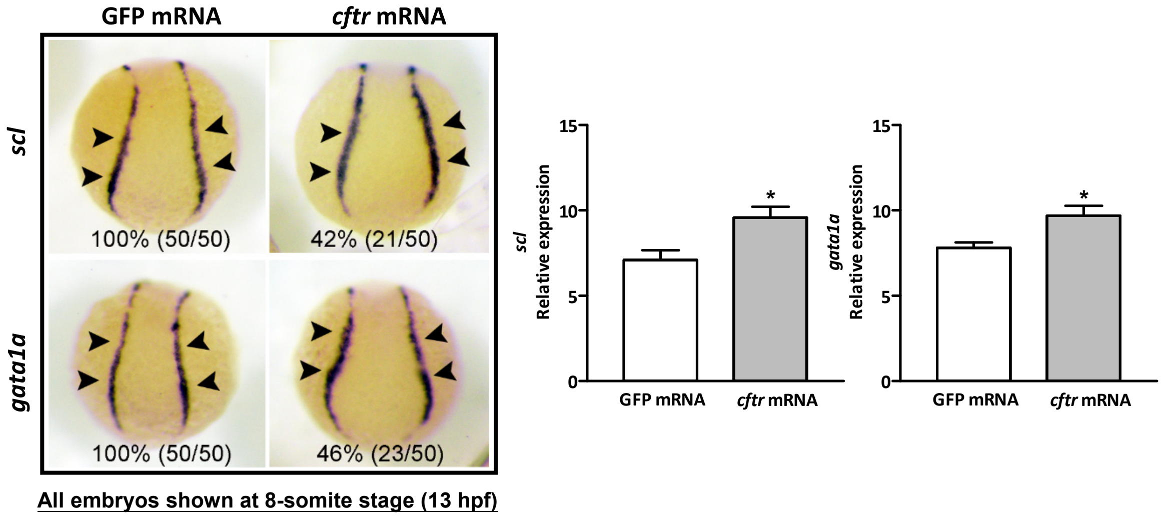

Supplementary figure 11

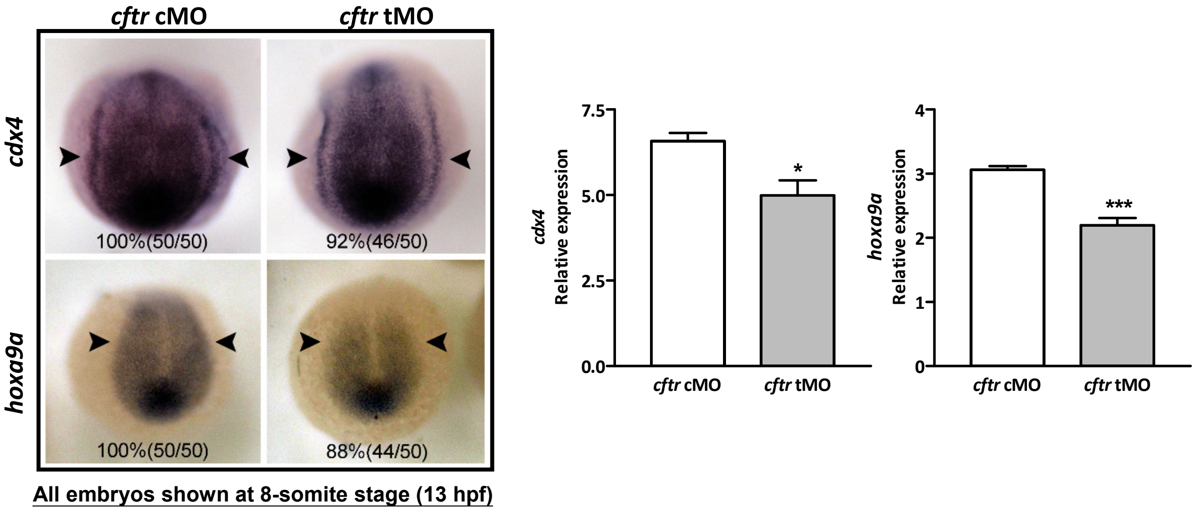

Supplementary figure 12

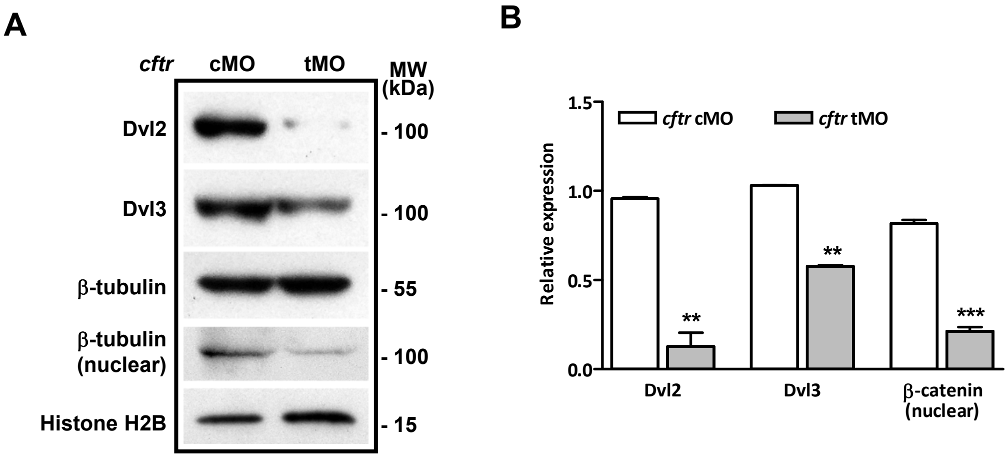

Supplementary figure 13

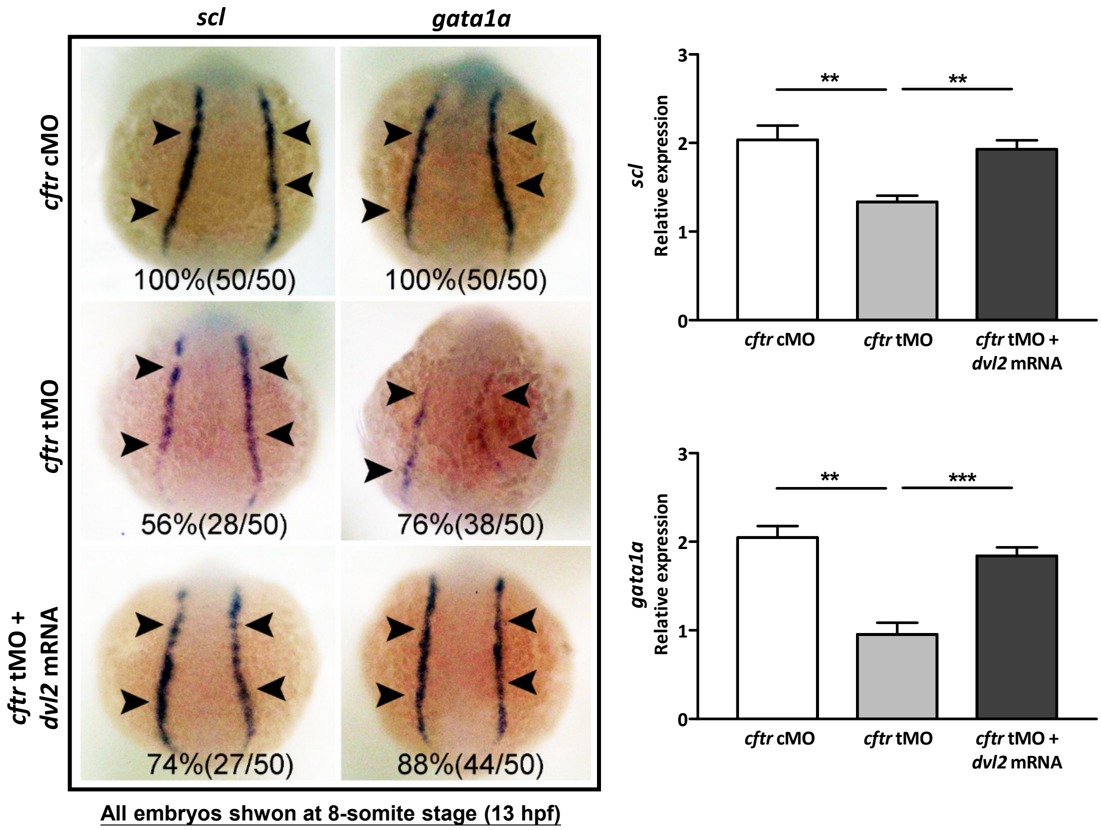

Supplementary figure 14

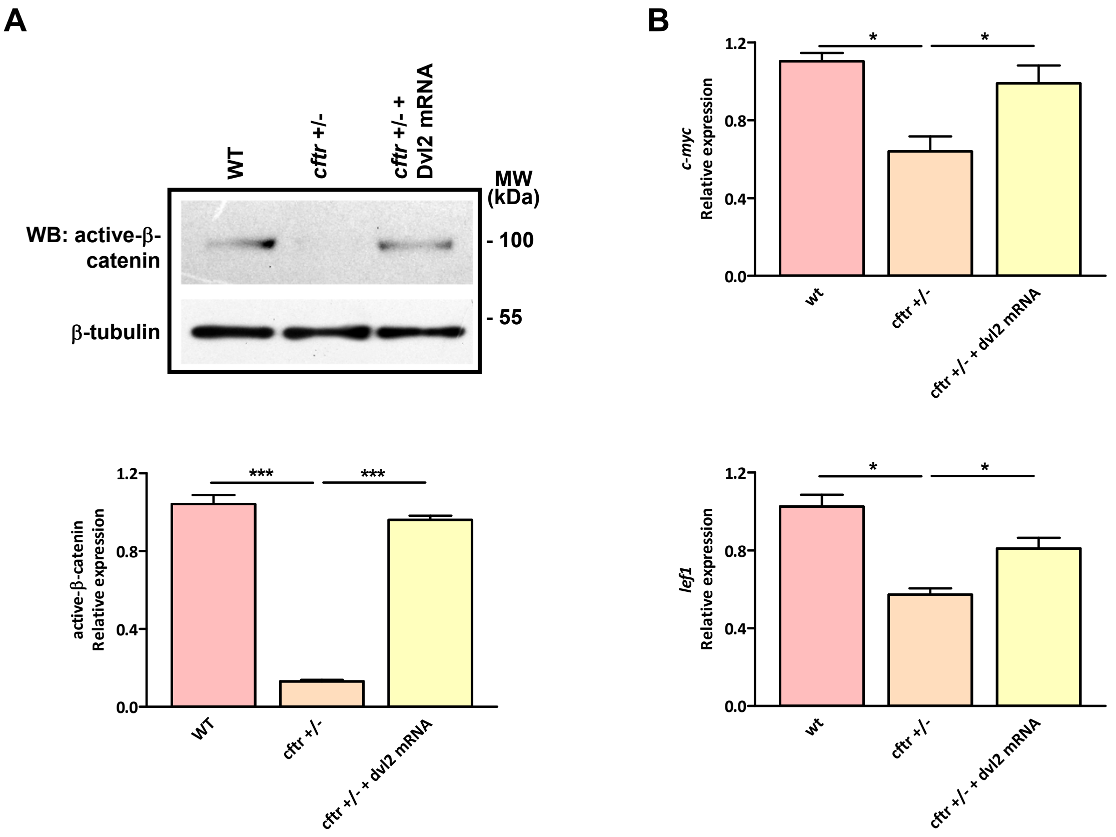

Supplementary figure 15

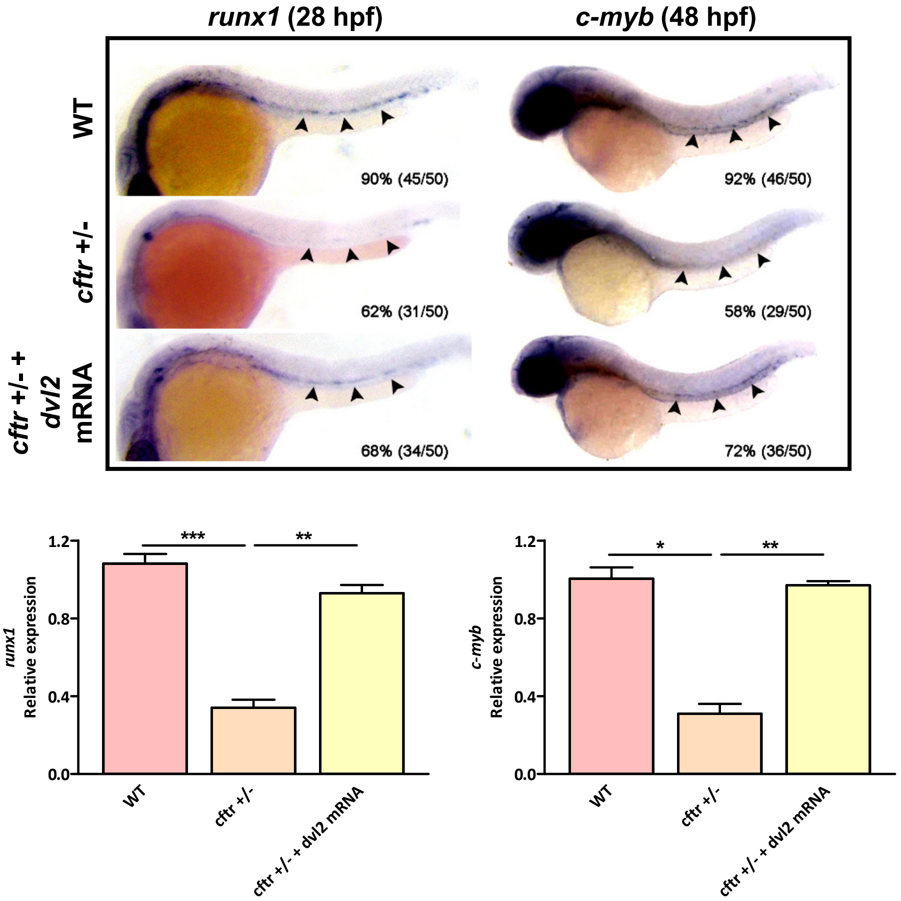

Supplementary figure 16

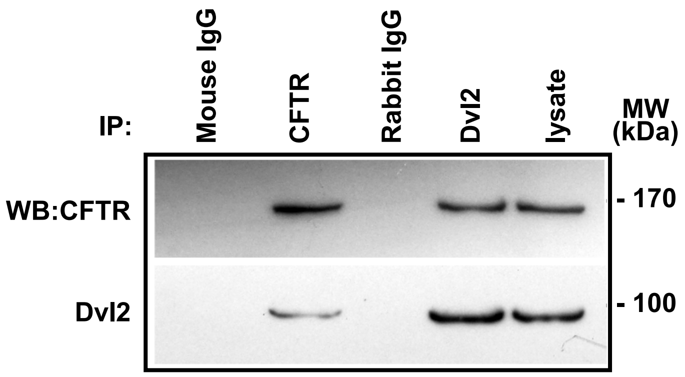

Supplementary figure 17

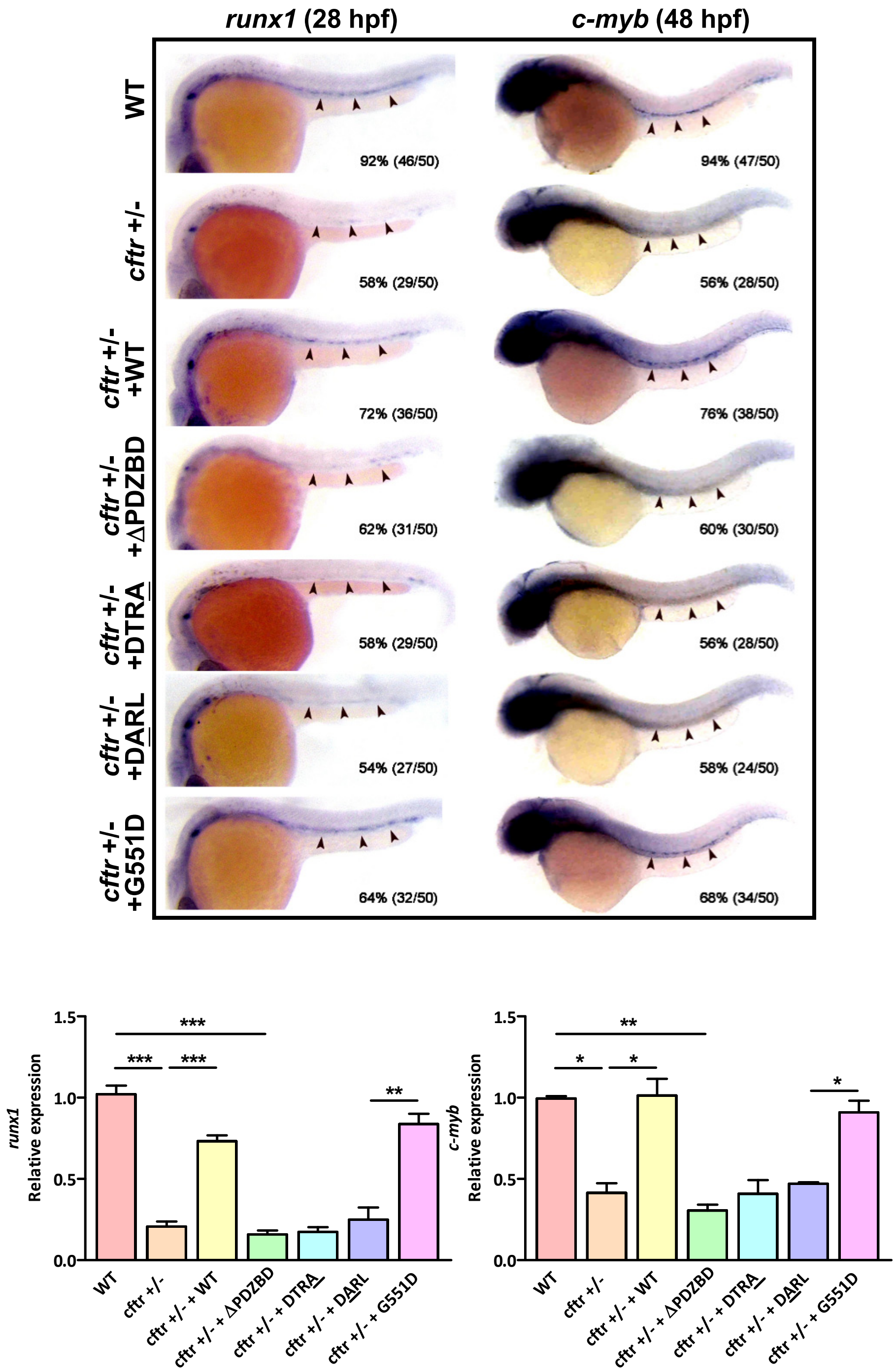

Supplementary figure 18

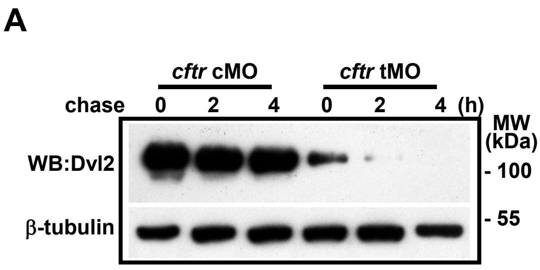

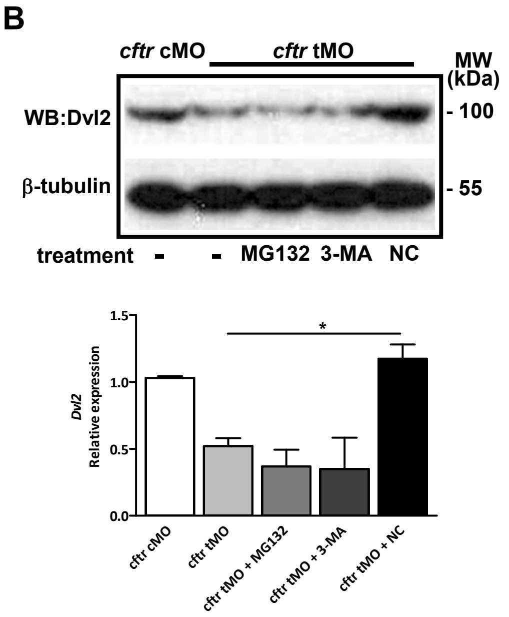

Supplementary figure 19
